# Variable selection in omics data: A practical evaluation of small sample sizes

**DOI:** 10.1371/journal.pone.0197910

**Published:** 2018-06-21

**Authors:** Alexander Kirpich, Elizabeth A. Ainsworth, Jessica M. Wedow, Jeremy R. B. Newman, George Michailidis, Lauren M. McIntyre

**Affiliations:** 1 Department of Biology, University of Florida, Gainesville, FL, United States of America; 2 Informatics Institute, University of Florida, Gainesville, FL, United States of America; 3 Department of Plant Biology, University of Illinois at Urbana-Champaign, Urbana, IL, United States of America; 4 USDA ARS Global Change and Photosynthesis Research Unit, Urbana, IL, United States of America; 5 Department of Statistics, University of Florida, Gainesville, FL, United States of America; 6 Genetics Institute, University of Florida, Gainesville, FL, United States of America; University of Arkansas for Medical Sciences, UNITED STATES

## Abstract

In omics experiments, variable selection involves a large number of metabolites/ genes and a small number of samples (the *n* < *p* problem). The ultimate goal is often the identification of one, or a few features that are different among conditions- a biomarker. Complicating biomarker identification, the *p* variables often contain a correlation structure due to the biology of the experiment making identifying causal compounds from correlated compounds difficult. Additionally, there may be elements in the experimental design (blocks, batches) that introduce structure in the data. While this problem has been discussed in the literature and various strategies proposed, the over fitting problems concomitant with such approaches are rarely acknowledged. Instead of viewing a single omics experiment as a definitive test for a biomarker, an unrealistic analytical goal, we propose to view such studies as screening studies where the goal of the study is to reduce the number of features present in the second round of testing, and to limit the Type II error. Using this perspective, the performance of LASSO, ridge regression and Elastic Net was compared with the performance of an ANOVA via a simulation study and two real data comparisons. Interestingly, a dramatic increase in the number of features had no effect on Type I error for the ANOVA approach. ANOVA, even without multiple test correction, has a low false positive rates in the scenarios tested. The Elastic Net has an inflated Type I error (from 10 to 50%) for small numbers of features which increases with sample size. The Type II error rate for the ANOVA is comparable or lower than that for the Elastic Net leading us to conclude that an ANOVA is an effective analytical tool for the initial screening of features in omics experiments.

## Introduction

In omics experiments, an analysis goal is the identification of features (metabolites or genes) that are different between treatment groups. The inspiration for this work is the analysis of untargeted metabolomics data, however the general results can be applied to other types of omics studies. In a typical metabolomics experiment the total number of samples is small and often limited by cost and processing time. The data collected are in the form of chromatograms, which are processed to select regions (peaks) that represent underlying chemical structures. Processing can be executed by proprietary instrument-specific software or with third party open source software, such as MZmine [1]. Selected peaks are quantified for each sample. This process has been well reviewed and well described [2] [3]. Peaks can be matched to metabolites/lipids and this identification process is its own area of research [4] [5]. In this work it is assumed, that quantified peaks (or gene expression levels) are the starting point for the analysis.

Typically, in omics experiments, the number of features is large in comparison to the number of samples and exceeds the number of available samples by orders of magnitude. Two alternative analysis paradigms are compared in this work. The first paradigm is based on classification approaches and compares the least absolute shrinkage and selection operator (LASSO), ridge regression and the generalization of these approaches—the Elastic Net feature selection methods, which accounts for the correlation structure among features. The second paradigm uses a linear models framework, where individual features are modeled separately ignoring the correlation structure among features, but allowing the incorporation of the experimental design structure directly into the model. By focusing on the “small” sample size *n* and “large” number of features *p* scenarios, these approaches are evaluated in settings likely to mimic those of the typical practitioner and on different datasets. Type I error and power have been estimated for the simulated data, to allow the practitioner to understand the performance of these approaches in real world settings.

Classification approaches have been deployed on subsets of original features selected after an initial “filtering” step. Prior comparisons deployed pre-screening of the original data, so that the final set of features available for further use and analysis was (much) smaller than the original set obtained from the instrument [6] [7] [8] [9] [10] [11]. Pre-screening using a *t*-test [6] [8] *t*-statistic scores [7] [12], Hardy-Weinberg equilibrium tests [9] and also non-statistical biological considerations [10] [6] have been proposed, with the subsequent application of statistical or machine learning methods to a subset of features. The rationale for the pre-screening of features is to aid in the efficient classification of samples into groups rather than feature selection [7] [13]. For example [6] after data pre-processing and pre-screening only 163 features remain out of ∼16,000 original data features. In the context of biomarker identification, feature selection rather than classification of samples is the goal. In the context of biomarker identification, the Type I and Type II errors of the entire process (pre-screening plus selection by machine learning) are unknown for real data. Here we focus on determining the Type I and Type II errors associated with feature selection using a single step.

In addition to the above studies which explicitly discuss “pre-screening” many methods developed for feature selection compare modern approaches using real data. In these comparisons, the prediction accuracy is used as the measure of performance since the Type I and Type II error are unknown in this setting. In a comparison of Multivariate Adaptive Regression Splines, Learning Ensemble (including bagging and boosting), Random Forest, Bayesian Moving Averaging, Stochastic Search Variable Selection, and Generalized Regularized Logistics Regression. The generalized regularized regression model (Elastic Net) had the highest predictive power [14]. A comparison of classifiers from 115 datasets found that Elastic Net was not different from bagging of *k*-nearest neighbors [15], support vector machine [16], and a 1-hidden layer neural network with sigmoid transfer function [17], [18]. In a recent comparison [19] of Boruta [20], the Vita method [21], recurrent relative variable importance [22], a parametric permutation approach [23] as well as recursive feature elimination (RFE) only permutation importance had some control of the Type I error. In direct comparisons of permutation importance and Elastic Net Random Forest was outperformed by Elastic Net [24].

In a simulation study performed by Acharjee et.al. [6] 100 samples were considered with 12 significant features out of 1000 comparing the performance of LASSO, Elastic Net, ridge regression, principal components regression (PCR), and other methods used for feature selection. The Elastic Net had the lowest mean squared error of prediction (MSEP) among the considered methods. LASSO, classification tree (CT), and linear discriminant analysis (LDA) were applied [8] for metabolic biosignature for Lyme disease prediction, where sample sizes were 202 and 259 for the treatment and control group, respectively. The number of features before and after pre-screening were 2262 and 95, respectively. Subgroups with sample sizes as small as 20 were evaluated. LASSO performed the best in terms of the receiver operating characteristic (ROC) curves for these data. Elastic Net had lower MSEP in comparison to support vector machines (SVM) and penalized logistic regression (PLSR) [7] [25]. LASSO has been shown to outperform OPLS-DA in feature selection [26] and Elastic Net is superior to stepwise selection [27].

LASSO, ridge regression and Elastic Net form a special class of penalized regression models. The first modeling approach with the penalty of that kind was ridge regression proposed by Hoerl and Kennard [28]. Due to the structure of the penalty ridge regression has a closed form solution for the standard linear models with normal errors and results in shrunk regression coefficients none of which is equal to zero. Thus, ridge regression can be used as a prediction tool but not as a feature selector directly. LASSO was introduced by Tibshirani [29]. While the LASSO method does not allow a closed form solutions it allows for variable selection. LASSO uses shrinkage to estimate which set of the regression coefficients have a value of zero and can therefore be eliminated. The LASSO method has limitations such as the number of variables that can be selected by the method has to be smaller or equal to the sample size *n*. The LASSO will also often select only a single feature in a set of highly correlated features [7].

The Elastic Net method introduced by Zou and Hastie [7] addressed the drawbacks of the LASSO and ridge regression methods, by creating a general framework and incorporated these two methods as special cases. The Elastic Net is a weighted combination of both LASSO and ridge regression penalties. The split between the penalties is controlled by the penalty split parameter *α* ∈ [0; 1] where *α* = 0 corresponds to ridge regression penalty and *α* = 1 corresponds to LASSO. Elastic Net was initially introduced for linear models with normal distribution of errors [7] and has later been extended to other types of models that included generalized linear models (such as logistic regression) and survival models together with efficient numerical computation algorithms [30] [31]. Elastic Net has also been shown to be a generalization of Support Vector Machines (SVM) [32] enabling some fast computational solutions developed for SVM to be applied to the Elastic Net. Elastic Net has been applied to the analysis of multiple real and simulated datasets, and has more than 2500 citations as of the writing of this manuscript. The computation algorithm proposed by Friedman et.al. [31] and the corresponding software package [33] has been used in this work.

**Fig 1:**
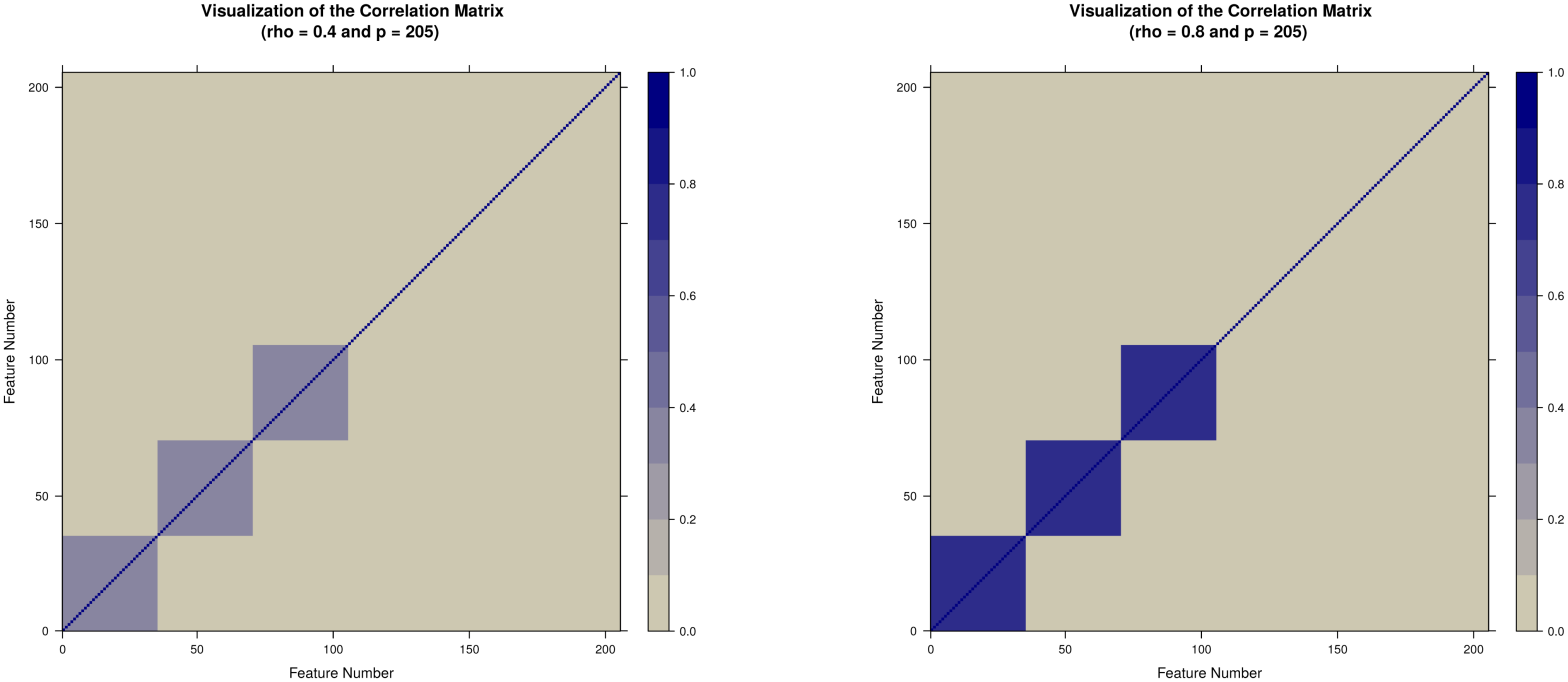
Visualization of power (left column) and Type I error (right column) estimates comparison between *p* = 205 (solid lines) and *p* = 2050 (dashed line) features for *ρ* = 0.4 and sample sizes *n* = 10 (top row), *n* = 50 (middle row), and *n* = 100 (bottom row). The value of the penalty split parameter *α* is plotted on the *x*-axis. Type I error and power estimates are plotted on *y*-axis for the values of *α* in the range of [0; 1] with 0.1 increments. In the left column power estimates are provided based on the four different features for each of the effect sizes (Δ_1_ = 0.2 is the red line, Δ_2_ = 0.5 is the blue line, and Δ_3_ = 0.8 is the green line). In the right column Type I error estimates are provided (beige lines) based on the random noise features together with a 0.05 threshold plotted as a purple dashed line. The vertical dashed line in the right column plots corresponds to penalty split value *α* = 0.5. The value of *α* = 0 corresponds to ridge regression and *α* = 1 corresponds to LASSO.

For omics data the number of samples *n* is often between 10 and 100. Such small sample sizes represent a challenge for the applied statistician. Despite this, little attention has been given to very small sample sizes. For example [31] the dataset with the smallest sample size considered was a leukemia dataset with *n* = 72 samples and *p* = 3571 features. The data were originally collected by Golub et.al. [34]. The authors report that *p* = 72 features were selected by LASSO, all features (*p* = 3571) were selected by ridge regression, and the Elastic Net with *α* = 0.2 selected values in between (Fig 1 [31]). In their simulation the smallest sample sizes considered was *n* = 100 [31]. Another simulation study comparing performance of ridge regression, LASSO and Elastic Net for small *n* and large *p* omics settings was conducted [35]. The authors examined sample sizes in the range *n* = 100, …, 1000 with a focus on prediction rather than feature selection. Ridge regression and Elastic Net were determined to outperform LASSO for prediction but the samples sizes smaller than 100 were not considered. Direct comparisons of LASSO, Elastic Net, classification and regression tree (CRT), random forest (RF), and *t*-tests for binary outcome and “non-big” data (*n* > *p*) using the simulation studies and psychiatric disorders data were performed [24]. The authors used sample sizes *n* = 200, 400 and *p* = 60 features for simulations which corresponds to the case of “non-big” data (*n* > *p*). For the real data considered in the paper the number of samples was *n* = 475 and the number of considered features was *p* = 44. The authors concluded that LASSO and Elastic Net provide superior performance for feature identification compared to other considered methods, and that a two-sided *t*-test performed well compared to LASSO and Elastic Net under certain scenarios [24].

In this work Elastic Net, LASSO and ridge regression are compared to an ANOVA approach for feature selection purposes with a focus on sample sizes less than 100. Simulation and two real data examples representing different aspects of omics studies are considered. In the first real dataset, a field experiment in maize, 24, B73 plants are evaluated in two conditions (ambient (n = 12) and elevated (n = 12) ozone). In the second experiment, 81 type 1 diabetics are examined in for gene expression differences in two different blood cell types (CD4+ and CD8+). In the maize experiment we expect very few differences among metabolites while in the diabetes data we expect large differences among the cell types. The goal in these and other similar experiments is to generate a list of features different between two experimental conditions for further evaluation; it is not to immediately generate a biomarker.

## Materials and methods

### Models

Given feature values *Y*_*ijk*_, where *i* is an indicator of the feature (*i* = 1, 2, …, *p*), *j* is an indicator of the sample (*j* = 1, 2, …, *n*), and *k* is an indicator of the treatment group (*k* = 1, 2, …, *m*), the primary interest is the identification of *i*-s such that corresponding features have different means for different treatment groups; i.e. those features where *μ*_*ik*_ ≠ *μ*_*ik*′_ for *k* ≠ *k*′.

In a linear model the *Y*_*ijk*_ are dependent outcomes. Without loss of generality the case of two groups (*m* = 2) is considered in this work. The formal model for feature *i* is either formulated as means model:

$$\begin{array}{c} \begin{array}{c} Y_{ijk}=\mu_{ik}+\epsilon_{ijk}\;\text{where}\;\epsilon_{ijk}\;∼iid\;N\left(0,\tau^{2}\right)\\ j=1,2,\ldots ,n\;\;\;k=1,2, \end{array} \end{array}$$ (1)

where *μ*_*ik*_ is the mean for feature *i* and group mean *k*. The alternative model formulation for feature *i* is the effects model:

$$\begin{array}{c} \begin{array}{c} Y_{ijk}=\mu_{i}+\nu_{ik}+\epsilon_{ijk}\;\text{where}\;\epsilon_{ijk}\;∼iid\;N\left(0,\tau^{2}\right)\\ j=1,2,\ldots ,n\;\;\;k=1,2, \end{array} \end{array}$$ (2)

where *μ*_*i*_ is the overall mean for feature *i* and *ν*_*ik*_ is the mean for feature *i* and group *k*. The required identifiability constraint for the effects model is $\sum_{k=1}^{2}\nu_{ik}=0$. The tests of hypothesis *H*_0_: *μ*_*i*1_ = *μ*_*i*2_ (or *H*_0_: *ν*_*i*1_ = *ν*_*i*2_) for each *i* with correlation among outcomes and a necessary adjustment for multiple testing to select features are used for comparison. The significance of difference for each feature *i* is determined by the corresponding ANOVA *F*-test. The ANOVA-based analysis was performed using R language and corresponding build-in function aov.

In the classification approach features ***Y***_*j*_ = (*Y*_1*j*_, *Y*_2*j*_, …, *Y*_*pj*_)^*T*^ are treated as predictors for the dependent binary random variable *K*_*j*_ that defines the group that sample *j* belongs to. The approach utilizes logistic regression with Elastic Net penalty proposed by Zou [7] and estimation algorithm proposed by Friedman et.al. [31]. The approach has been implemented in R package glmnet [31]. The detailed tutorial of the package has been provided online [36].

Let *G*_*k*_ represent the set of indexes of those samples that belong to group *k* and *I*_*jk*_ = *I*(*j* ∈ *G*_*k*_) be an indicator of sample *j* belonging to group *k*. For the two groups scenario the corresponding probabilities for sample *j* are modeled as

$$\begin{array}{c} \begin{array}{c} \mathrm{Pr}\left(K_{j}=2|Y_{j}\right)=\frac{\exp[\beta_{0}+\beta^{T}Y_{j}]}{1+\exp[\beta_{0}+\beta^{T}Y_{j}]}\\ \mathrm{Pr}\left(K_{j}=1|Y_{j}\right)=1-\mathrm{Pr}\left(K_{j}=2|Y_{j}\right)=\frac{1}{1+\exp[\beta_{0}+\beta^{T}Y_{j}]} \end{array} \end{array}$$ (3)

where ***β*** = (*β*_1_, *β*_2_, …, *β*_*p*_)^*T*^ is the set of logistic regression coefficients used for features. In combined notations the probability and the corresponding logged version have the form

$$\begin{array}{c} \begin{array}{c} \mathrm{Pr}\left(K_{j}=k|Y_{j}\right)=\frac{\exp[I_{j2}\left(\beta_{0}+\beta^{T}Y_{j}\right)]}{1+\exp[\beta_{0}+\beta^{T}Y_{j}]}\\ \log\left[\mathrm{Pr}\left(K_{j}=k|Y_{j}\right)\right]=I_{j2}\left(\beta_{0}+\beta^{T}Y_{j}\right)-\log[1+\exp[\beta_{0}+\beta^{T}Y_{j}]]. \end{array} \end{array}$$ (4)

The penalized log likelihood function for *n* samples have the from:

$$\begin{array}{c} \frac{1}{n}\sum_{j=1}^{n}[I_{j2}\left(\beta_{0}+\beta^{T}Y_{j}\right)-\log[1+\exp[\beta_{0}+\beta^{T}Y_{j}]]]+\lambda [[(1-\alpha )/2]{\left||\beta |\right|}_{2}^{2}+{\alpha ||\beta ||}_{1}] \end{array}$$ (5)

where ${\left||\beta |\right|}_{2}^{2}=\sum_{i=1}^{p}\beta_{i}^{2}$ and ${\left||\beta |\right|}_{1}=\sum_{i=1}^{p}\left|\beta_{i}\right|$. The second term in the penalized likelihood represents the Elastic Net penalty. The penalized likelihood is maximized numerically for parameters (*β*_0_, ***β***) for the given values of the penalty λ and split parameter *α*. The value *α* = 1 corresponds to LASSO penalty introduced by Tibshirani [29] and *α* = 0 corresponds to ridge regression penalty introduced by Hoerl and Kennard [28]. Due to the structure of the penalty outlined in (5) for all *α* > 0 some coefficient estimates ${\hat{\beta }}_{i}$ are equal to zero, and the procedure serves as a variable selector [7]. The value of penalty λ is often estimated via a cross-validation procedure. This may be problematic in small sample sizes [37] [38] motivating us to examine the behavior across a range of values for the split parameter *α*. In this classification framework, the problem transforms into variable selection problem with *p* variables *β*_*i*_ and binary categorical outcome *K*_*j*_. The Elastic Net approach directly accounts for the correlation among features.

### Simulation studies

Simulation studies were performed to compare, Elastic Net, ridge regression, and LASSO to ANOVA, in the identification of features with respect to the following questions: 1) What is the effect of sample size (*n*)? 2) What is the effect of the correlation structure among features? 3) What is the effect of increasing the number of features (*p*)? 4) What is the impact of effect size (Δ)?

For the control (*k* = 1) and treatment (*k* = 2) groups twenty scenarios were considered in the simulation study (Table 1) covering small (*p* = 205) and larger (*p* = 2050) numbers of features with medium (*ρ* = 0.4) and high (*ρ* = 0.8) correlation between the causal features and non-causal features as well as independent features representing stochastic noise for a range of sample sizes from *n* = 10 to 100 where *n*_1_ = *n*_2_ and *n*_1_+*n*_2_ = *n*.

**Table 1:** Summary of the simulation scenarios. For each scenario 1000 datasets were simulated. All together there were 20 scenarios considered.

Number of features (*p*)	Correlation (*ρ*)	Number of Samples (*n*)	Difference (Δ)
205	0.4	10	Δ_0_ = 0.0
2050	0.8	20	Δ_1_ = 0.2
		30	Δ_2_ = 0.5
		50	Δ_3_ = 0.8
		100	

All the samples were generated from the multivariate normal distribution (*MVN*) where the mean of each feature *μ*_*i*_ for *i* = 1, 2, …, *p* in the mean vector ***μ*** = (*μ*_1_, *μ*_2_, …, *μ*_*p*_)^*T*^ was preliminary independently generated from the univariate gamma distribution. The gamma distribution had the shape parameter *κ* = 50, the scale parameter *θ* = 1/50, and the density:

$$\begin{array}{c} f\left(\mu_{i}|\kappa ,\theta \right)=\frac{1}{\Gamma \left(\kappa \right)\theta^{\kappa }}\mu_{i}^{\kappa -1}e^{-\frac{\mu_{i}}{\theta }} \end{array}$$ (6)

where Γ(*κ*) is the gamma function. In the given parametrization the mean of the gamma distributions for each *μ*_*i*_ is equal to *κθ* = 1 and variance is equal to *κθ*^2^ = 1/50.

The variance-covariance matrix **Σ** used for the *MVN* simulation had a block diagonal structure with four independent blocks and diagonal variance values *σ*^2^ = 1. The first three blocks of **Σ** consisted of 35 (or 350) features with the fixed correlation value *ρ* between the elements within each block. The fourth block had 100 (or 1000) features generated independently from the normal distribution to represent stochastic noise likely present in most omics experiments. The parameters of the distribution were *MVN*(***μ***, **Σ**) where ***μ*** was the realization of the gamma distribution described in (6). Visualization of the correlation structure for *p* = 205 features is presented in Fig A in S1 Appendix. The structure for *p* = 2050 features is analogous. Twelve features were simulated with a difference in the means between the two treatment groups, four in each of the three correlated blocks with effect sizes Δ_1_ = 0.2, Δ_2_ = 0.5, and Δ_3_ = 0.8 for the first, the second, and the third block respectively. The effect size values Δ_1_ = 0.2, Δ_2_ = 0.5, and Δ_3_ = 0.8 according to Cohen [39] correspond to small, medium, and large effect sizes respectively. For each simulation scenario summarized in Table 1, 1000 datasets were generated and then analyzed with all methods. Simulation code is available in S1 File.

### Analysis methods

In the ANOVA approach the difference between the treatment groups was considered significant for a given feature if the corresponding one-way ANOVA *F*-test resulted in a nominal *p*-value smaller than 0.05. The ANOVA approach was implemented in R using the function aov from the build-in core package Stats. The false discovery rate (FDR) method proposed by Benjamini and Hochberg [40] was used to account for multiple testing. The FDR adjustment was implemented using the p.adjust function also from core package Stats and the difference between the treatment groups was considered significant if the adjusted *p*-value was smaller than thresholds that were used: 0.05 and 0.20.

For the Elastic Net approach the logistic regression with Elastic Net penalty was fit using the cv.glmnet() function [33]. The default number of folds (10) was used for cross-validation for all scenarios. The features selected by the method were the ones that had non-zero coefficients *β*_*i*_-s after the set was finalized. The Elastic Net method (5) depends on the choice of the penalty parameter *α* [7]. To investigate sensitivity of the results to values of *α*, analysis was performed on simulated data for *α* values 0 to 1, with 0.1 increment between the values. The *α*-sensitivity analysis was performed for each simulation scenario outlined in Table 1.

### Real datasets used for methods illustration

The maize data consist of *n* = 24 samples with *n*_1_ = 12 ambient and *n*_2_ = 12 ozone samples. The goal was to identify metabolites *i*, that were different between the ambient and ozone treatment groups (*k* = 1, 2). Ozone is a phytotoxic air pollutant that enters plants through the stomatal pores on their leaves, and ultimately results in reduced crop yields [41]. The goal of the study was to identify specific metabolites and metabolic pathways impacted by the air pollutant. Metabolomics data were generated by the South East Center for Integrated Matabolomics (SECIM) and have been deposited to the Metabolomics Workbench [42] with project ID PR000193. After QC filtering using information from the blanks [43] there were 986 metabolites in positive ion mode and 863 metabolites in negative ion mode. After the model (1) was fit, the FDR correction was applied separately to the positive and negative ion mode data.

A gene expression dataset for type 1 diabetes was also considered, consisting of Illumina HiSeq 2000 RNA sequencing data for three lymphocyte cell types (CD19+ B cells, CD4+ T cells, CD8+ T cells) from 81 subjects with type 1 diabetes [44]. Gene expression was quantified as measurements of exon abundance as previously described, and only the subset of 163,713 exons detected in all three cell types was analyzed [44]. For the purposes of illustrating the approach presented in this manuscript, only expression data from CD4+ and CD8+ T cells from the 79 individuals with complete data were analyzed to identify the set of differentially-expressed exons between CD4+ and CD8+ T cells in type 1 diabetes cases. Only those exons with average depths per nucleotide (APN) ≥5 were used for feature selection resulting in 8,268 exons that were present in all samples. Data are deposited in dbGaP [45] under accession number phs001426.v1.p1.

## Results

### Simulated data

**Fig 2:**
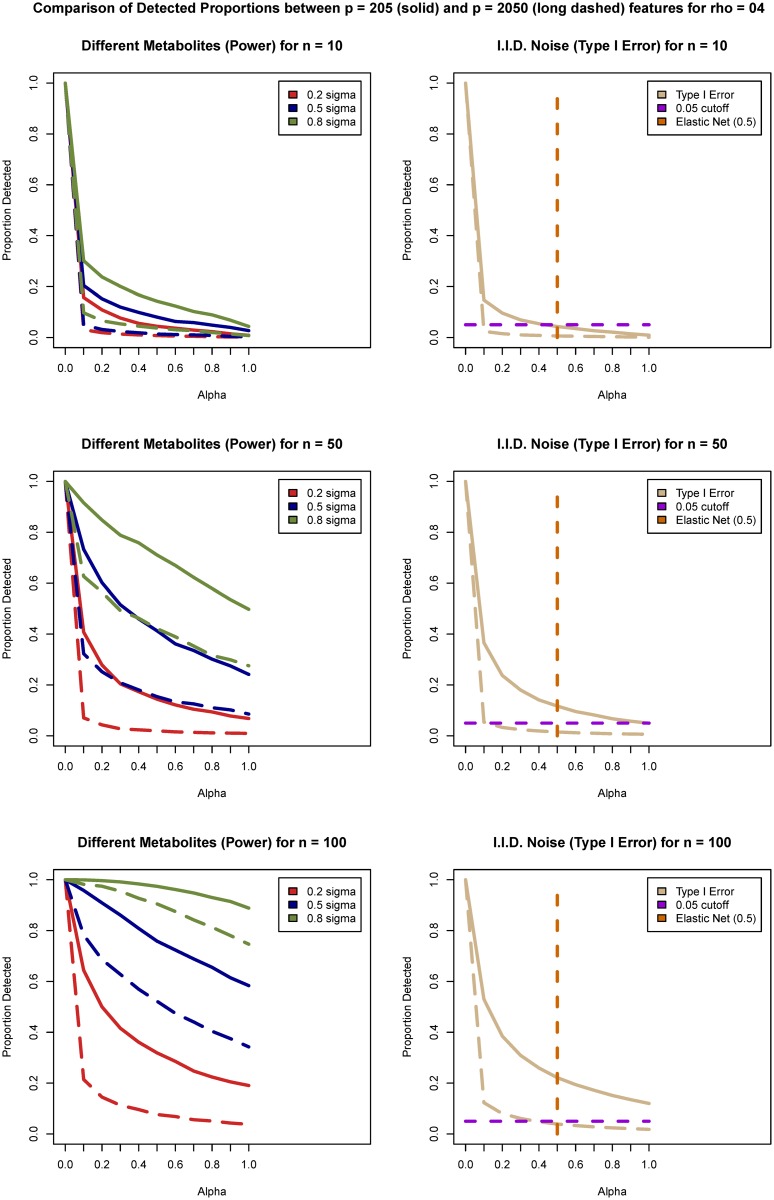
Visualization of power (left column) and Type I error (right column) estimates. Comparison between *p* = 205 (solid lines) and *p* = 2050 (dashed line) features for *ρ* = 0.8 and sample sizes *n* = 10 (top row), *n* = 50 (middle row), and *n* = 100 (bottom row). The value of the penalty split parameter *α* is plotted on the *x*-axis. Type I error and power estimates are plotted on *y*-axis for the values of *α* in the range of [0; 1] with 0.1 increments. In the left column power estimates are provided based on the four different features for each of the effect sizes (Δ_1_ = 0.2 is the red line, Δ_2_ = 0.5 is the blue line, and Δ_3_ = 0.8 is the green line). In the right column Type I error estimates are provided (beige lines) based on the random noise features together with a 0.05 threshold plotted as a purple dashed line. The vertical dashed line in the right column plots corresponds to penalty split value *α* = 0.5. The value of *α* = 0 corresponds to ridge regression and *α* = 1 corresponds to LASSO.

For the Elastic Net approach simulated data were evaluated across the range of possible values for the split parameter (Table 1). The parameter *α* that is responsible for a penalty split is referred to as a higher level parameter in the package documentation [36]. This *α*-sensitivity analysis was used to determine the values of *α* for use in comparison to the ANOVA approach. A subset of representative results is presented in Fig 1 (*ρ* = 0.4) and Fig 2 (*ρ* = 0.8). The complete set of results is provided in S1 Appendix Figs B–G. The summary statistics for the simulated data for sample sizes 10 and 100 are summarized in S1 Appendix Tables A–H. When fitting the Elastic Net model (5) there is also an option to cross validate not only the penalty value λ but also the penalty split parameter *α* [7]. When performing such cross validation the estimated value of *α* will be different for every dataset, which makes interpretation and justification of the specific penalty split parameter choice challenging. The possibility to over fit for small sample sizes should also be a concern [37] [38]. In this work a range of values for *α* was examined with the goal of selecting a single value.

For all simulation scenarios and for all values of *α* except 0 an increase in the effect size increased the power of detection. The increase in the sample size also increased the power of detection for all effect sizes and values of *α*. As the sample size increases the gain in power for small effect sizes is modest compared to large effect sizes for all methods. Power of detection decreased monotonically with increase in the penalty split value *α* from ridge regression (*α* = 0) to LASSO (*α* = 1). Ridge regression (*α* = 0) included all variables and resulted in estimates of power and Type I error of 1. LASSO (*α* = 1) had the lowest power of all values of *α* for each scenario and was in the range 0.7 − 0.8 only for the larger sample and effect sizes (Δ = 0.8 and *n* = 100).

For (*α* > 0) an increase in the sample size increased the Type I error. The increase was more pronounced for the smaller number of features (*p* = 205) than for the larger number of features (*p* = 2050). Type I error decreased monotonically with increase in the penalty split value *α* from ridge regression to LASSO. However, this is not a linear function. For ridge penalty (*α* = 0) coefficients of the correlated predictors are shrunk towards each other, for LASSO penalty (*α* = 1) only single coefficient of the correlated group selects one and discards the others [7]. The value *α* = 0.5 that was used for the analysis in this work tends to either select or not select the groups of correlated features together [36]. For the larger number of features (*p* = 2050) the steep reduction in Type I error occurs at much lower values of *α* than for the smaller number of features. The larger value of correlation values (*ρ* = 0.8 vs *ρ* = 0.4) increases Type I errors for larger sample sizes. For any value of *α*, Type I error was larger than nominal level 0.05 when the number of features was small (*p* = 205) and the sample size is *n* = 100. For the larger number of features (*p* = 2050) and small sample sizes (*n* = 10, 20, 30, 50) the Type I error was close to nominal level for *α* ∈ [0.5; 1] including for the LASSO. Based on these results *α* = 0.5 was considered a balanced representation of power and Type I error for the Elastic Net approach and was used for comparison with the ANOVA approach.

Type I error for the Elastic Net *α* = 0.5 and LASSO increased monotonically with the increase in the sample size but still stayed within the nominal level (0.05) for the considered sample sizes (*n* ≤ 100) if the number of features was larger (*p* = 2050). For the smaller number of features (*p* = 205) and sample sizes above fifty the error was above nominal level (0.05). To further investigate the behavior of Type I error based on the sample size for the larger number of features, the sizes *n* = 200, 500 and 1000 were considered for *p* = 2050 features and correlation *ρ* = 0.4. The results indicated that the Type I error kept increasing monotonically with the sample size increase for both LASSO and Elastic Net and were above the nominal level of 0.05 for sample size *n* = 200 for Elastic Net and for sample size *n* = 500 for LASSO.

The ANOVA tests were performed independently on each feature, the Type I error and the power of the tests were very similar regardless of the number of features and correlation between features. As expected, as sample size and effect size increased power increased. The power of ANOVA approach was in the range: 0.7 − 0.8 for sample sizes greater than *n* = 50 and effect size Δ = 0.8 *regardless* of the number of features. The results for Type I error were at the nominal level (0.05) for unadjusted ANOVA despite the multiple testing and as expected [40] were well below the nominal level of 0.2 after FDR adjustment. Indeed, after FDR adjustment at 0.20 the results were below 0.05.

For features simulated as correlated with differentially expressed features, but not themselves simulated to have a difference in the mean, the Elastic Net and LASSO approaches over selected features when the number of features was *p* = 205 and sample size was *n* ≥ 50. As expected, the performance of the ANOVA for these features mirrored the behavior of the Type I error for all sample sizes. Power estimates for sample sizes smaller than 50 were lower than 0.5 for small effect sizes for all tests. The increase in the effect size Δ increases power of the tests. Overall, LASSO performed worse than either Elastic Net or ANOVA (adjusted or unadjusted).

**Fig 3:**
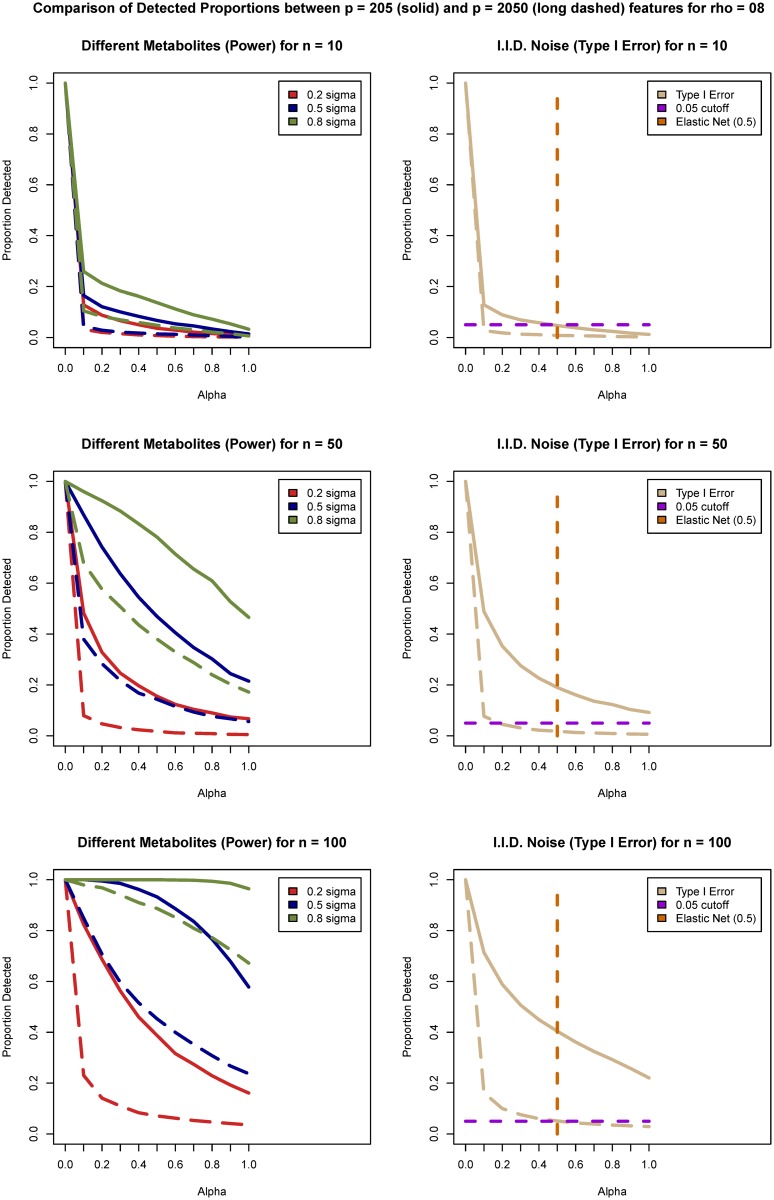
Visualization of power and Type I error estimates comparison for *p* = 205 features, correlation *ρ* = 0.4, and all sample sizes. Each row of the plots corresponds to a feature selection method. ANOVA FDR adjustment cutoff is 0.2. The value of the sample size (*n*) is displayed on the *x*-axis in all plots. The estimates of power and Type I error are provided on the *y*-axis. In the left column power estimates are provided based on the four different features for each of the effect sizes (Δ_1_ = 0.2 is the red line, Δ_2_ = 0.5 is the blue line, and Δ_3_ = 0.8 is the green line). In the right column Type I error estimates are provided (beige lines) based on the random noise features together with a 0.05 threshold plotted as a purple dashed line. In the middle column the proportions of non-different detected features within each block correlated to different ones for each of the blocks and corresponding effect sizes (Δ_1_ = 0.2 is the red line, Δ_2_ = 0.5 is the blue line, and Δ_3_ = 0.8 is the green line) are displayed.

**Fig 4:**
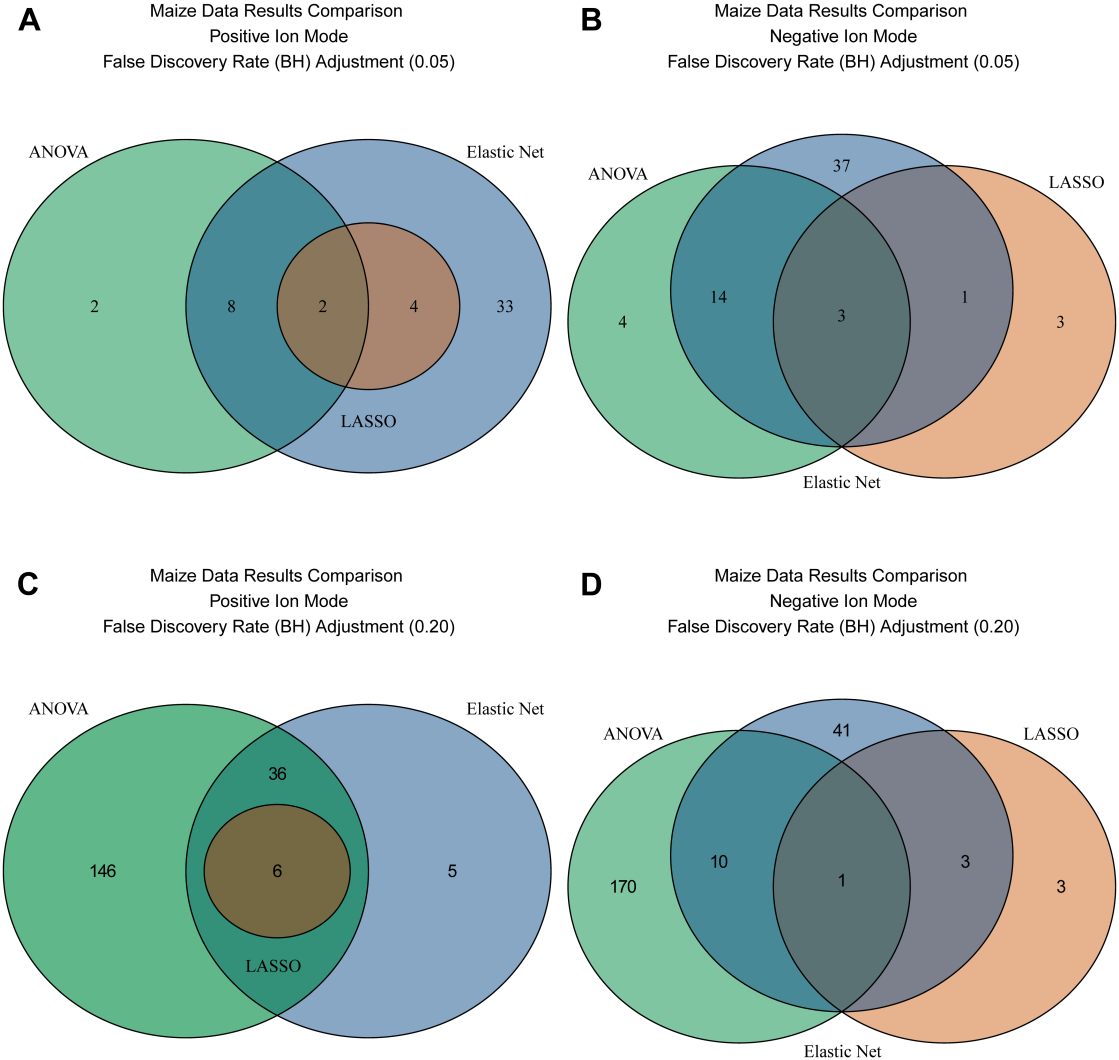
Visualization of power and Type I error estimates comparison for *p* = 2050 features, correlation *ρ* = 0.4, and all sample sizes. Each row of the plots corresponds to a feature selection method. ANOVA FDR adjustment cutoff is 0.2. The value of the sample size (*n*) is displayed on the *x*-axis in all plots. The estimates of power and Type I error are provided on the *y*-axis. In the left column power estimates are provided based on the four different features for each of the effect sizes (Δ_1_ = 0.2 is the red line, Δ_2_ = 0.5 is the blue line, and Δ_3_ = 0.8 is the green line). In the right column Type I error estimates are provided (beige lines) based on the random noise features together with a 0.05 threshold plotted as a purple dashed line. In the middle column the proportions of non-different detected features within each block correlated to different ones for each of the blocks and corresponding effect sizes (Δ_1_ = 0.2 is the red line, Δ_2_ = 0.5 is the blue line, and Δ_3_ = 0.8 is the green line) are displayed.

Of particular interest, the increase in the number of features from *p* = 205 to *p* = 2050 affected the Type I error and the power of any of the considered methods much less than the increase in number of samples from *n* = 10 to *n* = 100. This indicates that the number of features examined does not play as crucial a role as the correlation and dependency structure in the data and the sample size. The results for *ρ* = 0.4 are provided in Figs 3 and 4 and for *ρ* = 0.8 in S1 File Figs F and G.

### Real data

**Fig 5:**
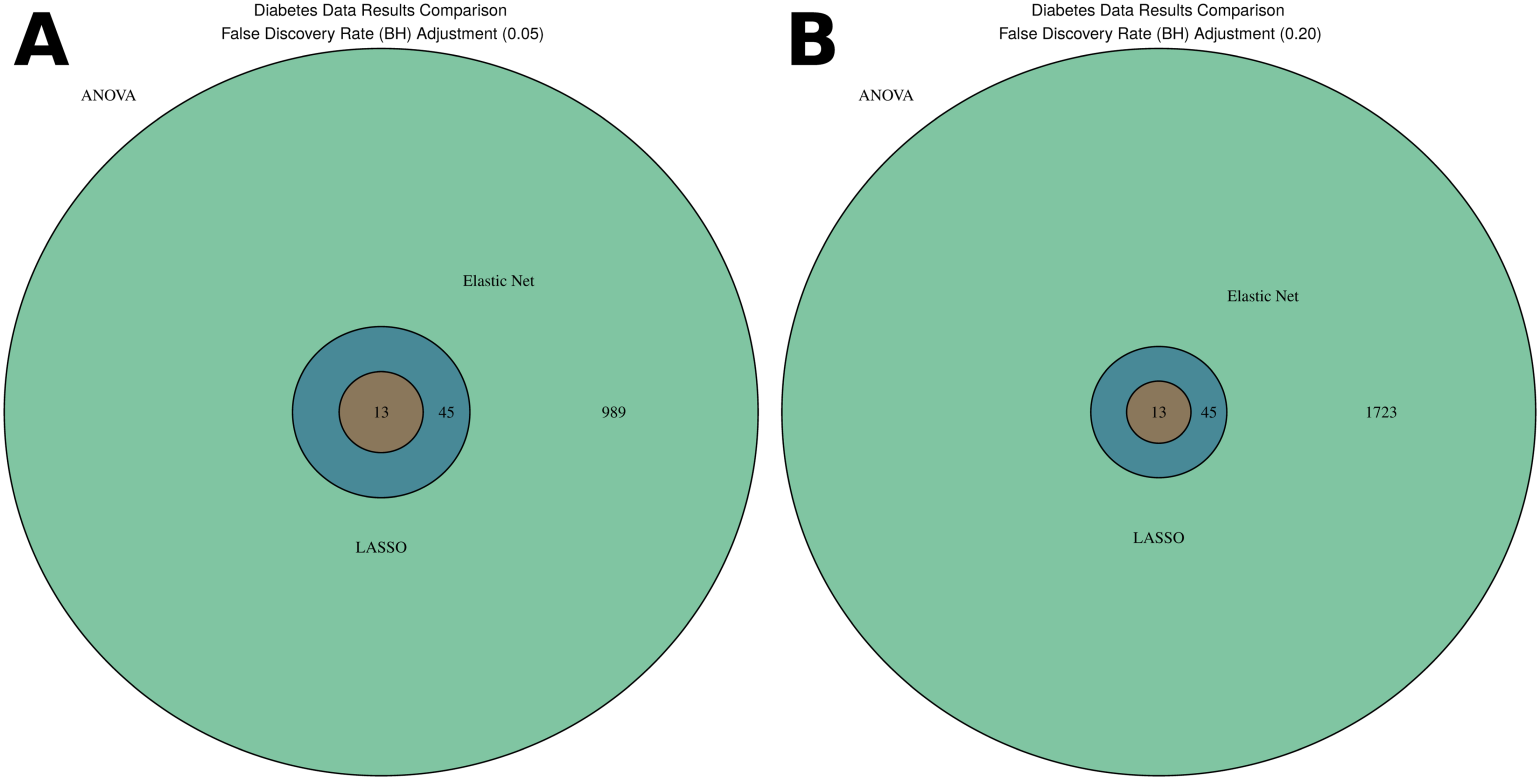
Venn diagrams depicting the results for the maize data. ANOVA (Green), Elastic Net (Blue) and LASSO (Brown) are compared. In Panel A the positive ion mode is shown with an FDR for the ANOVA of 0.05 while in Panel B the negative ion mode is show. Panel C is the positive ion mode with FDR of 0.2 and Panel D is the negative ion mode.

Venn diagrams (Fig 5, S1 Appendix Fig H) of the result comparison for different methods for maize data with FDR adjustment for ANOVA are presented in Fig 5 and without FDR adjustment in S1 Appendix Fig H. The results for the positive and negative ion modes were similar. When Type I error is minimized (FDR level 0.05) ANOVA selects comparable number of features to the Elastic Net and LASSO, but the three methods do not have perfect overlap between the set of features and none of the sets include the other. At an FDR level 0.2 the set of features selected by ANOVA does not include all the features selected by LASSO and Elastic Net. This results hold for both positive and negative ion modes. Interestingly, in the negative ion mode there were features selected by LASSO (*p* = 3) and not selected by Elastic Net. For positive ion model features selected by LASSO formed a proper subset of the features selected by Elastic Net.

In the diabetes data (Figs I and J in S1 Appendix) a set of *p* = 1781 exons was selected using the ANOVA method and FDR level 0.2 while the Elastic Net procedure selected a subset of 58 exons and the LASSO procedure selected 13 exons that formed a subset of Elastic Net selection. For the ANOVA method and FDR level 0.05 the set of *p* = 1047 exons were selected, and, again, the exons selected by LASSO formed a subset of exons selected by Elastic Net, which was in its turn the subset of the ANOVA features. The unadjusted ANOVA results are provided in S1 Appendix Fig I.

## Discussion

For omics data the identification of features that are potentially different among groups is a common analytical goal. Typically biologists view this initial experiment as a screening step although this is often not explicitly stated. It is not a realistic analytical goal to identify a biomarker in a single study, particularly a single study with sample sizes that are small. Many omics experiments have sample sizes in the tens. Further, there are many levels of structure in these data. There are biological correlations among the features examined due to the shared pathways that generate the observations and the experiments themselves often have a structure due to batch effects during data acquisition and potential covariates. For human studies, the initial sample collections may be complicated by patient characteristics such as age, sex or comorbidity. These analytic concerns should be considered in the evaluation of statistical methods for variable selection [46] [47] [48]. Here we explicitly consider the goal of the experiment to be the identification of a reasonable number of compounds that can be followed up. That is an explicit goal to minimize Type II error without undue inflation of the Type I error.

Methods have been developed for variable selection in omics data with the stated goal of finding the biomarker [6] [49] [50] [14]. However, it has been established that popular partial least squares discriminant analysis (PLS-DA) based approaches often overfit [51] [52]. Similarly machine learning approaches [53] [54] have been demonstrated to overfit for small samples. Elastic Net has been consistently found to perform well compared to other machine learning approaches [7] [6].

Elastic Net requires the selection of the penalty split *α* which can either be specified [36] [55] or, potentially, cross validated. There are several issues with cross validation in this context. First, cross validation for sample sizes less than 100 may be problematic due to poor performance [37] [38]. In this context cross-validation across the values of *α* in addition to the cross-validation over the values of λ is likely to perform poorly. Further, if cross-validation is performed for the penalty split parameter *α*, the analysis for each dataset will have a different value for $\hat{\alpha }$ which makes the direct comparison of the model challenging.

For small sample sizes when the number of features is also small, such as in a targeted panel of metabolites, the Elastic Net has an inflated Type I error. For larger numbers of features (*n* < *p*) the Elastic Net at small sample sizes (<100) has Type I error control, but has lower power than the uncorrected ANOVA. The uncorrected ANOVA does not have an inflation of the Type I error in these scenarios. Type I error inflation (overselection) for sample size of 50 has been reported for LASSO and other penalized approaches [56]. Ridge regression, will include all features in the prediction and has a Type I and Type II error of 1. The Elastic Net has also been shown to have a higher than nominal Type I error in studies with fairly large samples by omics standards (n = 500) [57].

Elastic Net has other distinct drawbacks in comparison to ANOVA as an initial screening tool. Elastic Net lacks an analytical solution [30] [31] making interpretation of the coefficient values based on values of the original measurement challenging. Further, some of the elements of the experiment may not be accounted for properly in normalization [58] [59] [60]. This may result in Elastic Net picking features that are (very) different between unaccounted for sources of variation (e.g. batch) and ignoring important but potentially smaller difference between treatments. This may explain why in the maize data there are features selected by Elastic Net that are not selected by the ANOVA approach. There is a possibility to include batch ID and other relevant variables as features that are subject to selection, but this is not expected to have the same effect as including covariates in an ANOVA model.

Often initial untargeted metabolomics studies are used to cast a broad net in identifying what set of compounds may be different among groups [61]. This is analogous to a screening study where the cost of the false negative should be weighed higher than the cost of the false positive, as subsequent experiments will remove the false positives. There is always a tradeoff [62] [63] [64]. As with screening studies, the follow up studies used to confirm a difference between groups are more expensive in labor, time and materials than the untargeted global approaches indicating that Type I error cannot be completely disregarded. The identification of groups of features that are differentially expressed from a shared pathway will provide insight into underlying biological processes [65] [66] [67]. Ideally correlated features that are truly differentially expressed should be selected and correlated features that are not different should not be selected. The ANOVA approach does not have an elevated Type I error rate among features correlated to those that are differentially expressed, compared to independent random noise, a desirable property.

The Type II error for the ANOVA approach was, in some circumstances, lower than the Elastic Net and LASSO. The conditions under which a two sample t-test can be used to extract all of the features that are different between conditions has recently been examined [68]. Fan and Fan prove that all features different between the two groups are selected with a probability of one (that is the probability of Type II errors goes to zero) assuming that most of the features are not different between the groups, Cramer’s condition holds and the variance for each feature is bounded away from zero. The diabetes data here clearly violate the first condition, as the number of genes different between the two conditions is large. However, in this case the ANOVA approach selected more features than the Elastic Net and the LASSO suggesting that the Type II error when the assumption of sparse differences is violated may not affect the Type II error.

The ultimate goal of a biomarker identification is to have one or a few biomarkers capable of predicting the treatment group. However, minimizing features at an early stage, using data from a single, small experiment risks the exclusion of the optimal biomarker. For biomarker identification to be successful, it should be thought of as a sequential screening procedure, a result of a preponderance of evidence over time. In the initial stages, selection of all of the significant correlated features (low Type II error), and none of the non-significant correlated features (low Type I error) allows the scientist to examine sets of significant features for biological insights. Enrichment analyses and other approaches can lend insights into the biological pathways and can help design subsequent experiments to measure those pathways more completely- for example developing a targeted panel of metabolites to be assayed in a large population based on the indication that the biological pathway is affected by the disease.

## Conclusion

An ANOVA is a simple technique, can account for a complex set of experimental design conditions [59], is likely to achieve a very low Type II error [68] and in the conditions studied here has lower Type I error than other more complex methods. For studies whose goal is to advance a set of features to the next round of testing for biological relevance, an ANOVA is an excellent choice.

## Supporting information

S1 Appendix The supplementary material contains tables and figures of the result summaries for the simulated and real data analysis that were not included in the main text due to the manuscript size limitations. (PDF)

S1 File The code for the simulations. (R)
