# Supplementary material for: Variable selection in omics data: A practical evaluation of small sample sizes
Source: PLoS One. 2018 Jun 21;13(6):e0197910. doi: 10.1371/journal.pone.0197910 (PMC6013185; doi:10.1371/journal.pone.0197910)
Supplement: S1 Appendix — (PDF) [file pone.0197910.s001.pdf]

S1 Appendix

Table A. The summary percentiles of the results across 1000 simulated datasets for $p = 205$ features, $\rho = 0.4$ and samples size $n = 10$. Each replicate was analyzed with ANOVA, Lasso, Elastic Net and ridge regression. For each replicate simulation the 205 features were simulated according to the set of parameters in Table 1. In particular, here the first three blocks of $\mathbf{\Sigma}$ consisted of 35 features with the fixed correlation value ρ between the elements within each block, each of these blocks had four features with effect sizes Δ_1, Δ_2, and Δ_3 and 31 features with no effect size, representing random noise correlated to features with signal. The fourth block had 100 features generated independently from the normal distribution to represent stochastic noise likely present in most omics experiments. For features that are simulated as random noise, selecting that feature would be a false positive. For a feature simulated as different selection is a true positive or an estimate of power. For each method the selection of each feature in each of the 1000 iterations was recorded. For ANOVA feature selection was based on the p-value, for LASSO/ridge regression and Elastic Net features were selected if they had a non-zero coefficient. The proportion of times that a feature was detected over the 1000 replicates is calculated. For a random noise feature this is then the estimate of the Type I error. As each simulation has multiple features in each category we report the estimated quantiles across features using the `R` function `quantiles`. For ridge regression there is no shrinkage and so all features are always selected.

Type	0%	2.4%	25%	50%	75%	97.6%	100%
LASSO Bl. 1 (1-4) Δ_1	0.007	0.007	0.007	0.008	0.011	0.013	0.013
LASSO Bl. 1 (5-35) Same	0.001	0.001	0.006	0.007	0.009	0.013	0.014
LASSO Bl. 2 (36-39) Δ_2	0.018	0.019	0.023	0.027	0.029	0.030	0.030
LASSO Bl. 2 (40-70) Same	0.003	0.004	0.006	0.009	0.012	0.015	0.017
LASSO Bl. 3 (71-74) Δ_3	0.039	0.039	0.042	0.044	0.045	0.049	0.049
LASSO Bl. 3 (75-105) Same	0.005	0.005	0.006	0.008	0.009	0.013	0.014
LASSO Bl. 4 (106-205) Same	0.001	0.004	0.007	0.009	0.012	0.018	0.020
Elastic Net Bl. 1 (1-4) Δ_1	0.036	0.036	0.038	0.044	0.050	0.051	0.051
Elastic Net Bl. 1 (5-35) Same	0.030	0.031	0.038	0.041	0.044	0.050	0.051
Elastic Net Bl. 2 (36-39) Δ_2	0.066	0.067	0.071	0.080	0.089	0.094	0.095
Elastic Net Bl. 2 (40-70) Same	0.031	0.035	0.040	0.043	0.048	0.052	0.052
Elastic Net Bl. 3 (71-74) Δ_3	0.135	0.135	0.139	0.142	0.150	0.164	0.166
Elastic Net Bl. 3 (75-105) Same	0.023	0.026	0.036	0.043	0.046	0.049	0.050
Elastic Net Bl. 4 (106-205) Same	0.030	0.032	0.038	0.043	0.047	0.053	0.059
ANOVA Bl. 1 (1-4) Δ_1	0.059	0.059	0.062	0.065	0.068	0.070	0.070
ANOVA Bl. 1 (5-35) Same	0.042	0.042	0.049	0.051	0.056	0.060	0.061
ANOVA Bl. 2 (36-39) Δ_2	0.094	0.094	0.095	0.100	0.105	0.108	0.108
ANOVA Bl. 2 (40-70) Same	0.041	0.043	0.050	0.053	0.057	0.064	0.065
ANOVA Bl. 3 (71-74) Δ_3	0.187	0.188	0.194	0.196	0.204	0.221	0.223
ANOVA Bl. 3 (75-105) Same	0.033	0.039	0.046	0.049	0.052	0.060	0.066
ANOVA Bl. 4 (106-205) Same	0.036	0.039	0.046	0.050	0.054	0.063	0.067
ANOVA (FDR-BH) Bl. 1 (1-4) Δ_1	0.003	0.003	0.003	0.003	0.004	0.008	0.008
ANOVA (FDR-BH) Bl. 1 (5-35) Same	0.000	0.001	0.002	0.002	0.004	0.005	0.006
ANOVA (FDR-BH) Bl. 2 (36-39) Δ_2	0.006	0.006	0.009	0.010	0.011	0.012	0.012
ANOVA (FDR-BH) Bl. 2 (40-70) Same	0.000	0.000	0.001	0.002	0.004	0.006	0.007
ANOVA (FDR-BH) Bl. 3 (71-74) Δ_3	0.014	0.014	0.015	0.016	0.017	0.021	0.021
ANOVA (FDR-BH) Bl. 3 (75-105) Same	0.000	0.001	0.002	0.004	0.004	0.006	0.006
ANOVA (FDR-BH) Bl. 4 (106-205) Same	0.000	0.000	0.001	0.002	0.003	0.005	0.006

Table B. The summary percentiles of the results across 1000 simulated datasets for $p = 205$ features, $\rho = 0.4$ and samples size $n = 100$. Each replicate was analyzed with ANOVA, Lasso, Elastic Net and ridge regression. For each replicate simulation the 205 features were simulated according to the set of parameters in Table 1. In particular, here the first three blocks of $\mathbf{\Sigma}$ consisted of 35 features with the fixed correlation value ρ between the elements within each block, each of these blocks had four features with effect sizes Δ_1, Δ_2, and Δ_3 and 31 features with no effect size, representing random noise correlated to features with signal. The fourth block had 100 features generated independently from the normal distribution to represent stochastic noise likely present in most omics experiments. For features that are simulated as random noise, selecting that feature would be a false positive. For a feature simulated as different selection is a true positive or an estimate of power. For each method the selection of each feature in each of the 1000 iterations was recorded. For ANOVA feature selection was based on the p-value, for LASSO/ridge regression and Elastic Net features were selected if they had a non-zero coefficient. The proportion of times that a feature was detected over the 1000 replicates is calculated. For a random noise feature this is then the estimate of the Type I error. As each simulation has multiple features in each category we report the estimated quantiles across features using the `R` function `quantiles`. For ridge regression there is no shrinkage and so all features are always selected.

Type	0%	2.4%	25%	50%	75%	97.6%	100%
LASSO Bl. 1 (1-4) Δ_1	0.182	0.182	0.184	0.184	0.186	0.192	0.193
LASSO Bl. 1 (5-35) Same	0.051	0.053	0.062	0.065	0.069	0.083	0.086
LASSO Bl. 2 (36-39) Δ_2	0.567	0.567	0.570	0.576	0.582	0.586	0.587
LASSO Bl. 2 (40-70) Same	0.075	0.076	0.084	0.088	0.092	0.110	0.117
LASSO Bl. 3 (71-74) Δ_3	0.885	0.885	0.887	0.888	0.891	0.896	0.897
LASSO Bl. 3 (75-105) Same	0.165	0.169	0.184	0.192	0.197	0.209	0.209
LASSO Bl. 4 (106-205) Same	0.091	0.099	0.113	0.118	0.126	0.142	0.147
Elastic Net Bl. 1 (1-4) Δ_1	0.301	0.301	0.306	0.314	0.322	0.329	0.330
Elastic Net Bl. 1 (5-35) Same	0.124	0.125	0.136	0.144	0.150	0.163	0.167
Elastic Net Bl. 2 (36-39) Δ_2	0.749	0.749	0.750	0.756	0.764	0.776	0.777
Elastic Net Bl. 2 (40-70) Same	0.153	0.156	0.163	0.173	0.178	0.200	0.205
Elastic Net Bl. 3 (71-74) Δ_3	0.966	0.967	0.972	0.974	0.976	0.979	0.979
Elastic Net Bl. 3 (75-105) Same	0.235	0.248	0.267	0.277	0.286	0.296	0.296
Elastic Net Bl. 4 (106-205) Same	0.188	0.197	0.211	0.220	0.229	0.255	0.264
ANOVA Bl. 1 (1-4) Δ_1	0.159	0.160	0.170	0.176	0.179	0.184	0.185
ANOVA Bl. 1 (5-35) Same	0.038	0.039	0.044	0.048	0.051	0.059	0.065
ANOVA Bl. 2 (36-39) Δ_2	0.661	0.663	0.683	0.691	0.696	0.705	0.706
ANOVA Bl. 2 (40-70) Same	0.045	0.046	0.051	0.056	0.059	0.069	0.075
ANOVA Bl. 3 (71-74) Δ_3	0.976	0.976	0.977	0.978	0.979	0.980	0.980
ANOVA Bl. 3 (75-105) Same	0.032	0.033	0.042	0.047	0.053	0.056	0.057
ANOVA Bl. 4 (106-205) Same	0.035	0.037	0.047	0.052	0.056	0.064	0.070
ANOVA (FDR-BH) Bl. 1 (1-4) Δ_1	0.051	0.052	0.057	0.060	0.061	0.062	0.062
ANOVA (FDR-BH) Bl. 1 (5-35) Same	0.003	0.004	0.007	0.010	0.010	0.015	0.017
ANOVA (FDR-BH) Bl. 2 (36-39) Δ_2	0.396	0.397	0.406	0.412	0.416	0.422	0.423
ANOVA (FDR-BH) Bl. 2 (40-70) Same	0.006	0.007	0.009	0.012	0.014	0.016	0.017
ANOVA (FDR-BH) Bl. 3 (71-74) Δ_3	0.876	0.876	0.880	0.882	0.883	0.887	0.887
ANOVA (FDR-BH) Bl. 3 (75-105) Same	0.004	0.004	0.006	0.008	0.010	0.014	0.015
ANOVA (FDR-BH) Bl. 4 (106-205) Same	0.003	0.004	0.007	0.008	0.011	0.014	0.014

Table C. The summary percentiles of the results across 1000 simulated datasets for $p = 2050$ features, $\rho = 0.4$ and samples size $n = 10$. Each replicate was analyzed with ANOVA, Lasso, Elastic Net and ridge regression. For each replicate simulation the 2050 features were simulated according to the set of parameters in Table 1. In particular, here the first three blocks of $\mathbf{\Sigma}$ consisted of 350 features with the fixed correlation value ρ between the elements within each block, each of these blocks had four features with effect sizes Δ_1, Δ_2, and Δ_3 and 346 features with no effect size, representing random noise correlated to features with signal. The fourth block had 1000 features generated independently from the normal distribution to represent stochastic noise likely present in most omics experiments. For features that are simulated as random noise, selecting that feature would be a false positive. For a feature simulated as different selection is a true positive or an estimate of power. For each method the selection of each feature in each of the 1000 iterations was recorded. For ANOVA feature selection was based on the p-value, for LASSO/ridge regression and Elastic Net features were selected if they had a non-zero coefficient. The proportion of times that a feature was detected over the 1000 replicates is calculated. For a random noise feature this is then the estimate of the Type I error. As each simulation has multiple features in each category we report the estimated quantiles across features using the `R` function `quantiles`. For ridge regression there is no shrinkage and so all features are always selected.

Type	0%	2.4%	25%	50%	75%	97.6%	100%
LASSO Bl. 1 (1-4) Δ_1	0.000	0.000	0.001	0.002	0.002	0.002	0.002
LASSO Bl. 1 (5-350) Same	0.000	0.000	0.000	0.001	0.001	0.003	0.004
LASSO Bl. 2 (351-354) Δ_2	0.001	0.001	0.002	0.003	0.004	0.004	0.004
LASSO Bl. 2 (355-700) Same	0.000	0.000	0.000	0.001	0.001	0.003	0.004
LASSO Bl. 3 (701-704) Δ_3	0.007	0.007	0.007	0.008	0.008	0.008	0.008
LASSO Bl. 3 (705-1050) Same	0.000	0.000	0.000	0.001	0.001	0.003	0.004
LASSO Bl. 4 (1051-2050) Same	0.000	0.000	0.000	0.001	0.002	0.004	0.006
Elastic Net Bl. 1 (1-4) Δ_1	0.007	0.007	0.007	0.008	0.008	0.009	0.009
Elastic Net Bl. 1 (5-350) Same	0.001	0.002	0.005	0.007	0.009	0.012	0.017
Elastic Net Bl. 2 (351-354) Δ_2	0.012	0.012	0.013	0.014	0.014	0.016	0.016
Elastic Net Bl. 2 (355-700) Same	0.001	0.003	0.005	0.007	0.008	0.012	0.015
Elastic Net Bl. 3 (701-704) Δ_3	0.035	0.035	0.036	0.037	0.038	0.040	0.040
Elastic Net Bl. 3 (705-1050) Same	0.001	0.002	0.005	0.006	0.008	0.012	0.014
Elastic Net Bl. 4 (1051-2050) Same	0.000	0.002	0.005	0.006	0.008	0.012	0.014
ANOVA Bl. 1 (1-4) Δ_1	0.051	0.051	0.054	0.058	0.062	0.069	0.070
ANOVA Bl. 1 (5-350) Same	0.025	0.038	0.047	0.051	0.056	0.064	0.073
ANOVA Bl. 2 (351-354) Δ_2	0.097	0.098	0.109	0.113	0.113	0.114	0.114
ANOVA Bl. 2 (355-700) Same	0.036	0.039	0.046	0.050	0.055	0.064	0.069
ANOVA Bl. 3 (701-704) Δ_3	0.192	0.193	0.198	0.204	0.214	0.228	0.230
ANOVA Bl. 3 (705-1050) Same	0.033	0.037	0.045	0.050	0.054	0.062	0.068
ANOVA Bl. 4 (1051-2050) Same	0.031	0.037	0.046	0.050	0.055	0.064	0.074
ANOVA (FDR-BH) Bl. 1 (1-4) Δ_1	0.001	0.001	0.001	0.001	0.002	0.003	0.003
ANOVA (FDR-BH) Bl. 1 (5-350) Same	0.000	0.000	0.000	0.001	0.001	0.003	0.003
ANOVA (FDR-BH) Bl. 2 (351-354) Δ_2	0.001	0.001	0.001	0.001	0.002	0.003	0.003
ANOVA (FDR-BH) Bl. 2 (355-700) Same	0.000	0.000	0.000	0.001	0.001	0.002	0.004
ANOVA (FDR-BH) Bl. 3 (701-704) Δ_3	0.002	0.002	0.003	0.003	0.004	0.005	0.005
ANOVA (FDR-BH) Bl. 3 (705-1050) Same	0.000	0.000	0.000	0.000	0.001	0.002	0.003
ANOVA (FDR-BH) Bl. 4 (1051-2050) Same	0.000	0.000	0.000	0.000	0.000	0.002	0.003

Table D. The summary percentiles of the results across 1000 simulated datasets for $p = 2050$ features, $\rho = 0.4$ and samples size $n = 100$. Each replicate was analyzed with ANOVA, Lasso, Elastic Net and ridge regression. For each replicate simulation the 2050 features were simulated according to the set of parameters in Table 1. In particular, here the first three blocks of $\mathbf{\Sigma}$ consisted of 350 features with the fixed correlation value ρ between the elements within each block, each of these blocks had four features with effect sizes Δ_1, Δ_2, and Δ_3 and 346 features with no effect size, representing random noise correlated to features with signal. The fourth block had 1000 features generated independently from the normal distribution to represent stochastic noise likely present in most omics experiments. For features that are simulated as random noise, selecting that feature would be a false positive. For a feature simulated as different selection is a true positive or an estimate of power. For each method the selection of each feature in each of the 1000 iterations was recorded. For ANOVA feature selection was based on the p-value, for LASSO/ridge regression and Elastic Net features were selected if they had a non-zero coefficient. The proportion of times that a feature was detected over the 1000 replicates is calculated. For a random noise feature this is then the estimate of the Type I error. As each simulation has multiple features in each category we report the estimated quantiles across features using the `R` function `quantiles`. For ridge regression there is no shrinkage and so all features are always selected.

Type	0%	2.4%	25%	50%	75%	97.6%	100%
LASSO Bl. 1 (1-4) Δ_1	0.031	0.031	0.033	0.040	0.048	0.052	0.052
LASSO Bl. 1 (5-350) Same	0.000	0.002	0.004	0.005	0.007	0.010	0.015
LASSO Bl. 2 (351-354) Δ_2	0.327	0.328	0.338	0.344	0.349	0.354	0.355
LASSO Bl. 2 (355-700) Same	0.000	0.001	0.004	0.006	0.008	0.012	0.015
LASSO Bl. 3 (701-704) Δ_3	0.749	0.749	0.749	0.751	0.755	0.761	0.762
LASSO Bl. 3 (705-1050) Same	0.006	0.009	0.014	0.016	0.019	0.025	0.026
LASSO Bl. 4 (1051-2050) Same	0.004	0.010	0.015	0.018	0.021	0.027	0.034
Elastic Net Bl. 1 (1-4) Δ_1	0.063	0.063	0.066	0.080	0.094	0.095	0.095
Elastic Net Bl. 1 (5-350) Same	0.003	0.007	0.011	0.013	0.016	0.021	0.023
Elastic Net Bl. 2 (351-354) Δ_2	0.499	0.500	0.512	0.522	0.530	0.535	0.536
Elastic Net Bl. 2 (355-700) Same	0.005	0.008	0.012	0.014	0.017	0.023	0.026
Elastic Net Bl. 3 (701-704) Δ_3	0.898	0.899	0.904	0.908	0.911	0.915	0.915
Elastic Net Bl. 3 (705-1050) Same	0.013	0.017	0.023	0.027	0.031	0.037	0.041
Elastic Net Bl. 4 (1051-2050) Same	0.020	0.027	0.036	0.040	0.044	0.052	0.063
ANOVA Bl. 1 (1-4) Δ_1	0.138	0.139	0.146	0.151	0.162	0.186	0.188
ANOVA Bl. 1 (5-350) Same	0.033	0.036	0.045	0.050	0.053	0.062	0.067
ANOVA Bl. 2 (351-354) Δ_2	0.673	0.674	0.679	0.688	0.700	0.714	0.715
ANOVA Bl. 2 (355-700) Same	0.030	0.035	0.044	0.048	0.053	0.060	0.065
ANOVA Bl. 3 (701-704) Δ_3	0.971	0.971	0.975	0.978	0.979	0.980	0.980
ANOVA Bl. 3 (705-1050) Same	0.032	0.040	0.048	0.053	0.058	0.067	0.074
ANOVA Bl. 4 (1051-2050) Same	0.024	0.037	0.045	0.050	0.055	0.064	0.075
ANOVA (FDR-BH) Bl. 1 (1-4) Δ_1	0.005	0.005	0.006	0.007	0.008	0.009	0.009
ANOVA (FDR-BH) Bl. 1 (5-350) Same	0.000	0.000	0.001	0.002	0.002	0.004	0.006
ANOVA (FDR-BH) Bl. 2 (351-354) Δ_2	0.124	0.124	0.126	0.130	0.145	0.173	0.176
ANOVA (FDR-BH) Bl. 2 (355-700) Same	0.000	0.000	0.000	0.001	0.001	0.003	0.004
ANOVA (FDR-BH) Bl. 3 (701-704) Δ_3	0.620	0.621	0.625	0.632	0.637	0.638	0.638
ANOVA (FDR-BH) Bl. 3 (705-1050) Same	0.000	0.000	0.001	0.002	0.003	0.004	0.007
ANOVA (FDR-BH) Bl. 4 (1051-2050) Same	0.000	0.000	0.000	0.000	0.001	0.003	0.005

Table E. The summary percentiles of the results across 1000 simulated datasets for $p = 205$ features, $\rho = 0.8$ and samples size $n = 10$. Each replicate was analyzed with ANOVA, Lasso, Elastic Net and ridge regression. For each replicate simulation the 205 features were simulated according to the set of parameters in Table 1. In particular, here the first three blocks of $\mathbf{\Sigma}$ consisted of 35 features with the fixed correlation value ρ between the elements within each block, each of these blocks had four features with effect sizes Δ_1, Δ_2, and Δ_3 and 31 features with no effect size, representing random noise correlated to features with signal. The fourth block had 100 features generated independently from the normal distribution to represent stochastic noise likely present in most omics experiments. For features that are simulated as random noise, selecting that feature would be a false positive. For a feature simulated as different selection is a true positive or an estimate of power. For each method the selection of each feature in each of the 1000 iterations was recorded. For ANOVA feature selection was based on the p-value, for LASSO/ridge regression and Elastic Net features were selected if they had a non-zero coefficient. The proportion of times that a feature was detected over the 1000 replicates is calculated. For a random noise feature this is then the estimate of the Type I error. As each simulation has multiple features in each category we report the estimated quantiles across features using the `R` function `quantiles`. For ridge regression there is no shrinkage and so all features are always selected.

Type	0%	2.4%	25%	50%	75%	97.6%	100%
LASSO Bl. 1 (1-4) Δ_1	0.005	0.005	0.006	0.007	0.007	0.008	0.008
LASSO Bl. 1 (5-35) Same	0.002	0.002	0.004	0.004	0.007	0.011	0.012
LASSO Bl. 2 (36-39) Δ_2	0.011	0.011	0.012	0.014	0.015	0.016	0.016
LASSO Bl. 2 (40-70) Same	0.002	0.002	0.003	0.004	0.005	0.007	0.008
LASSO Bl. 3 (71-74) Δ_3	0.025	0.025	0.030	0.032	0.034	0.036	0.036
LASSO Bl. 3 (75-105) Same	0.000	0.001	0.002	0.003	0.004	0.006	0.006
LASSO Bl. 4 (106-205) Same	0.004	0.006	0.010	0.012	0.015	0.022	0.023
Elastic Net Bl. 1 (1-4) Δ_1	0.035	0.035	0.035	0.036	0.039	0.043	0.044
Elastic Net Bl. 1 (5-35) Same	0.020	0.021	0.028	0.031	0.034	0.037	0.038
Elastic Net Bl. 2 (36-39) Δ_2	0.064	0.064	0.065	0.067	0.070	0.071	0.071
Elastic Net Bl. 2 (40-70) Same	0.022	0.023	0.026	0.030	0.031	0.038	0.040
Elastic Net Bl. 3 (71-74) Δ_3	0.124	0.125	0.133	0.137	0.140	0.144	0.145
Elastic Net Bl. 3 (75-105) Same	0.018	0.018	0.022	0.025	0.028	0.032	0.034
Elastic Net Bl. 4 (106-205) Same	0.027	0.037	0.043	0.047	0.052	0.058	0.064
ANOVA Bl. 1 (1-4) Δ_1	0.055	0.055	0.057	0.059	0.060	0.062	0.062
ANOVA Bl. 1 (5-35) Same	0.041	0.042	0.048	0.051	0.054	0.059	0.060
ANOVA Bl. 2 (36-39) Δ_2	0.094	0.094	0.098	0.102	0.106	0.107	0.107
ANOVA Bl. 2 (40-70) Same	0.045	0.046	0.051	0.054	0.058	0.063	0.065
ANOVA Bl. 3 (71-74) Δ_3	0.189	0.189	0.191	0.194	0.197	0.203	0.204
ANOVA Bl. 3 (75-105) Same	0.033	0.035	0.038	0.042	0.045	0.052	0.052
ANOVA Bl. 4 (106-205) Same	0.039	0.040	0.047	0.050	0.055	0.064	0.067
ANOVA (FDR-BH) Bl. 1 (1-4) Δ_1	0.013	0.013	0.013	0.014	0.014	0.014	0.014
ANOVA (FDR-BH) Bl. 1 (5-35) Same	0.008	0.009	0.011	0.013	0.014	0.017	0.018
ANOVA (FDR-BH) Bl. 2 (36-39) Δ_2	0.008	0.008	0.010	0.010	0.011	0.011	0.011
ANOVA (FDR-BH) Bl. 2 (40-70) Same	0.009	0.009	0.011	0.012	0.014	0.016	0.016
ANOVA (FDR-BH) Bl. 3 (71-74) Δ_3	0.017	0.017	0.019	0.020	0.022	0.023	0.023
ANOVA (FDR-BH) Bl. 3 (75-105) Same	0.005	0.005	0.008	0.008	0.010	0.012	0.013
ANOVA (FDR-BH) Bl. 4 (106-205) Same	0.000	0.000	0.002	0.003	0.004	0.006	0.006

Table F. The summary percentiles of the results across 1000 simulated datasets for $p = 205$ features, $\rho = 0.8$ and samples size $n = 100$. Each replicate was analyzed with ANOVA, Lasso, Elastic Net and ridge regression. For each replicate simulation the 205 features were simulated according to the set of parameters in Table 1. In particular, here the first three blocks of $\mathbf{\Sigma}$ consisted of 35 features with the fixed correlation value ρ between the elements within each block, each of these blocks had four features with effect sizes Δ_1, Δ_2, and Δ_3 and 31 features with no effect size, representing random noise correlated to features with signal. The fourth block had 100 features generated independently from the normal distribution to represent stochastic noise likely present in most omics experiments. For features that are simulated as random noise, selecting that feature would be a false positive. For a feature simulated as different selection is a true positive or an estimate of power. For each method the selection of each feature in each of the 1000 iterations was recorded. For ANOVA feature selection was based on the p-value, for LASSO/ridge regression and Elastic Net features were selected if they had a non-zero coefficient. The proportion of times that a feature was detected over the 1000 replicates is calculated. For a random noise feature this is then the estimate of the Type I error. As each simulation has multiple features in each category we report the estimated quantiles across features using the `R` function `quantiles`. For ridge regression there is no shrinkage and so all features are always selected.

Type	0%	2.4%	25%	50%	75%	97.6%	100%
LASSO Bl. 1 (1-4) Δ_1	0.152	0.152	0.154	0.158	0.164	0.169	0.170
LASSO Bl. 1 (5-35) Same	0.018	0.019	0.025	0.028	0.030	0.037	0.038
LASSO Bl. 2 (36-39) Δ_2	0.524	0.527	0.556	0.575	0.590	0.605	0.607
LASSO Bl. 2 (40-70) Same	0.075	0.079	0.096	0.102	0.108	0.120	0.120
LASSO Bl. 3 (71-74) Δ_3	0.959	0.959	0.962	0.964	0.966	0.970	0.970
LASSO Bl. 3 (75-105) Same	0.297	0.297	0.309	0.318	0.331	0.345	0.345
LASSO Bl. 4 (106-205) Same	0.192	0.195	0.212	0.222	0.230	0.247	0.252
Elastic Net Bl. 1 (1-4) Δ_1	0.368	0.369	0.380	0.390	0.395	0.396	0.396
Elastic Net Bl. 1 (5-35) Same	0.076	0.081	0.090	0.099	0.106	0.115	0.119
Elastic Net Bl. 2 (36-39) Δ_2	0.912	0.913	0.926	0.932	0.937	0.941	0.942
Elastic Net Bl. 2 (40-70) Same	0.235	0.241	0.257	0.266	0.276	0.294	0.297
Elastic Net Bl. 3 (71-74) Δ_3	0.999	0.999	1.000	1.000	1.000	1.000	1.000
Elastic Net Bl. 3 (75-105) Same	0.471	0.472	0.484	0.492	0.498	0.521	0.538
Elastic Net Bl. 4 (106-205) Same	0.365	0.370	0.391	0.402	0.412	0.428	0.438
ANOVA Bl. 1 (1-4) Δ_1	0.159	0.159	0.163	0.166	0.168	0.169	0.169
ANOVA Bl. 1 (5-35) Same	0.042	0.043	0.050	0.054	0.058	0.063	0.065
ANOVA Bl. 2 (36-39) Δ_2	0.679	0.680	0.694	0.704	0.710	0.716	0.717
ANOVA Bl. 2 (40-70) Same	0.041	0.041	0.046	0.049	0.053	0.054	0.054
ANOVA Bl. 3 (71-74) Δ_3	0.974	0.974	0.976	0.978	0.979	0.981	0.981
ANOVA Bl. 3 (75-105) Same	0.039	0.040	0.050	0.054	0.057	0.064	0.065
ANOVA Bl. 4 (106-205) Same	0.039	0.041	0.047	0.050	0.054	0.061	0.063
ANOVA (FDR-BH) Bl. 1 (1-4) Δ_1	0.051	0.051	0.052	0.055	0.058	0.062	0.062
ANOVA (FDR-BH) Bl. 1 (5-35) Same	0.016	0.017	0.020	0.022	0.024	0.027	0.028
ANOVA (FDR-BH) Bl. 2 (36-39) Δ_2	0.425	0.425	0.428	0.433	0.438	0.440	0.440
ANOVA (FDR-BH) Bl. 2 (40-70) Same	0.011	0.011	0.013	0.015	0.016	0.020	0.020
ANOVA (FDR-BH) Bl. 3 (71-74) Δ_3	0.863	0.864	0.868	0.870	0.871	0.871	0.871
ANOVA (FDR-BH) Bl. 3 (75-105) Same	0.011	0.011	0.015	0.017	0.018	0.021	0.021
ANOVA (FDR-BH) Bl. 4 (106-205) Same	0.004	0.004	0.007	0.009	0.011	0.014	0.017

Table G. The summary percentiles of the results across 1000 simulated datasets for $p = 2050$ features, $\rho = 0.8$ and samples size $n = 10$. Each replicate was analyzed with ANOVA, Lasso, Elastic Net and ridge regression. For each replicate simulation the 2050 features were simulated according to the set of parameters in Table 1. In particular, here the first three blocks of $\mathbf{\Sigma}$ consisted of 350 features with the fixed correlation value ρ between the elements within each block, each of these blocks had four features with effect sizes Δ_1, Δ_2, and Δ_3 and 346 features with no effect size, representing random noise correlated to features with signal. The fourth block had 1000 features generated independently from the normal distribution to represent stochastic noise likely present in most omics experiments. For features that are simulated as random noise, selecting that feature would be a false positive. For a feature simulated as different selection is a true positive or an estimate of power. For each method the selection of each feature in each of the 1000 iterations was recorded. For ANOVA feature selection was based on the p-value, for LASSO/ridge regression and Elastic Net features were selected if they had a non-zero coefficient. The proportion of times that a feature was detected over the 1000 replicates is calculated. For a random noise feature this is then the estimate of the Type I error. As each simulation has multiple features in each category we report the estimated quantiles across features using the `R` function `quantiles`. For ridge regression there is no shrinkage and so all features are always selected.

Type	0%	2.4%	25%	50%	75%	97.6%	100%
LASSO Bl. 1 (1-4) Δ_1	0.000	0.000	0.000	0.000	0.001	0.001	0.001
LASSO Bl. 1 (5-350) Same	0.000	0.000	0.000	0.000	0.001	0.002	0.002
LASSO Bl. 2 (351-354) Δ_2	0.000	0.000	0.001	0.001	0.001	0.001	0.001
LASSO Bl. 2 (355-700) Same	0.000	0.000	0.000	0.000	0.000	0.002	0.003
LASSO Bl. 3 (701-704) Δ_3	0.004	0.004	0.005	0.006	0.007	0.008	0.008
LASSO Bl. 3 (705-1050) Same	0.000	0.000	0.000	0.000	0.001	0.002	0.003
LASSO Bl. 4 (1051-2050) Same	0.000	0.000	0.001	0.001	0.002	0.004	0.006
Elastic Net Bl. 1 (1-4) Δ_1	0.005	0.005	0.006	0.008	0.008	0.010	0.010
Elastic Net Bl. 1 (5-350) Same	0.001	0.001	0.004	0.005	0.007	0.010	0.013
Elastic Net Bl. 2 (351-354) Δ_2	0.010	0.010	0.011	0.014	0.018	0.020	0.020
Elastic Net Bl. 2 (355-700) Same	0.000	0.000	0.002	0.003	0.004	0.008	0.009
Elastic Net Bl. 3 (701-704) Δ_3	0.035	0.036	0.043	0.048	0.051	0.053	0.053
Elastic Net Bl. 3 (705-1050) Same	0.000	0.001	0.003	0.004	0.006	0.008	0.011
Elastic Net Bl. 4 (1051-2050) Same	0.001	0.004	0.007	0.008	0.010	0.014	0.020
ANOVA Bl. 1 (1-4) Δ_1	0.061	0.061	0.062	0.064	0.066	0.073	0.074
ANOVA Bl. 1 (5-350) Same	0.036	0.041	0.050	0.053	0.057	0.065	0.067
ANOVA Bl. 2 (351-354) Δ_2	0.086	0.086	0.090	0.094	0.098	0.099	0.099
ANOVA Bl. 2 (355-700) Same	0.029	0.032	0.037	0.040	0.044	0.051	0.057
ANOVA Bl. 3 (701-704) Δ_3	0.203	0.203	0.205	0.209	0.214	0.219	0.220
ANOVA Bl. 3 (705-1050) Same	0.032	0.037	0.044	0.048	0.051	0.060	0.068
ANOVA Bl. 4 (1051-2050) Same	0.032	0.037	0.045	0.050	0.054	0.065	0.081
ANOVA (FDR-BH) Bl. 1 (1-4) Δ_1	0.010	0.010	0.011	0.012	0.012	0.013	0.013
ANOVA (FDR-BH) Bl. 1 (5-350) Same	0.005	0.006	0.009	0.010	0.011	0.014	0.015
ANOVA (FDR-BH) Bl. 2 (351-354) Δ_2	0.007	0.007	0.008	0.008	0.008	0.008	0.008
ANOVA (FDR-BH) Bl. 2 (355-700) Same	0.001	0.003	0.005	0.006	0.007	0.009	0.010
ANOVA (FDR-BH) Bl. 3 (701-704) Δ_3	0.006	0.006	0.008	0.008	0.008	0.010	0.010
ANOVA (FDR-BH) Bl. 3 (705-1050) Same	0.002	0.004	0.006	0.007	0.008	0.009	0.011
ANOVA (FDR-BH) Bl. 4 (1051-2050) Same	0.000	0.000	0.000	0.001	0.002	0.003	0.005

Table H. The summary percentiles of the results across 1000 simulated datasets for $p = 2050$ features, $\rho = 0.8$ and samples size $n = 100$. Each replicate was analyzed with ANOVA, Lasso, Elastic Net and ridge regression. For each replicate simulation the 2050 features were simulated according to the set of parameters in Table 1. In particular, here the first three blocks of $\mathbf{\Sigma}$ consisted of 350 features with the fixed correlation value ρ between the elements within each block, each of these blocks had four features with effect sizes Δ_1, Δ_2, and Δ_3 and 346 features with no effect size, representing random noise correlated to features with signal. The fourth block had 1000 features generated independently from the normal distribution to represent stochastic noise likely present in most omics experiments. For features that are simulated as random noise, selecting that feature would be a false positive. For a feature simulated as different selection is a true positive or an estimate of power. For each method the selection of each feature in each of the 1000 iterations was recorded. For ANOVA feature selection was based on the p-value, for LASSO/ridge regression and Elastic Net features were selected if they had a non-zero coefficient. The proportion of times that a feature was detected over the 1000 replicates is calculated. For a random noise feature this is then the estimate of the Type I error. As each simulation has multiple features in each category we report the estimated quantiles across features using the `R` function `quantiles`. For ridge regression there is no shrinkage and so all features are always selected.

Type	0%	2.4%	25%	50%	75%	97.6%	100%
LASSO Bl. 1 (1-4) Δ_1	0.031	0.031	0.035	0.036	0.037	0.039	0.039
LASSO Bl. 1 (5-350) Same	0.000	0.000	0.000	0.001	0.002	0.004	0.004
LASSO Bl. 2 (351-354) Δ_2	0.233	0.233	0.234	0.242	0.249	0.253	0.253
LASSO Bl. 2 (355-700) Same	0.000	0.000	0.000	0.001	0.002	0.004	0.005
LASSO Bl. 3 (701-704) Δ_3	0.659	0.660	0.666	0.671	0.677	0.684	0.685
LASSO Bl. 3 (705-1050) Same	0.007	0.009	0.014	0.017	0.019	0.026	0.028
LASSO Bl. 4 (1051-2050) Same	0.014	0.019	0.026	0.030	0.033	0.040	0.046
Elastic Net Bl. 1 (1-4) Δ_1	0.065	0.065	0.070	0.074	0.078	0.078	0.078
Elastic Net Bl. 1 (5-350) Same	0.000	0.000	0.002	0.003	0.004	0.007	0.009
Elastic Net Bl. 2 (351-354) Δ_2	0.432	0.433	0.443	0.453	0.460	0.465	0.465
Elastic Net Bl. 2 (355-700) Same	0.000	0.000	0.002	0.004	0.005	0.009	0.010
Elastic Net Bl. 3 (701-704) Δ_3	0.880	0.881	0.885	0.887	0.888	0.890	0.890
Elastic Net Bl. 3 (705-1050) Same	0.016	0.019	0.024	0.028	0.031	0.039	0.044
Elastic Net Bl. 4 (1051-2050) Same	0.028	0.037	0.046	0.051	0.056	0.066	0.072
ANOVA Bl. 1 (1-4) Δ_1	0.137	0.137	0.137	0.138	0.140	0.147	0.148
ANOVA Bl. 1 (5-350) Same	0.028	0.034	0.040	0.043	0.047	0.054	0.056
ANOVA Bl. 2 (351-354) Δ_2	0.694	0.695	0.702	0.708	0.711	0.713	0.713
ANOVA Bl. 2 (355-700) Same	0.034	0.038	0.045	0.048	0.052	0.060	0.065
ANOVA Bl. 3 (701-704) Δ_3	0.969	0.969	0.974	0.975	0.976	0.978	0.978
ANOVA Bl. 3 (705-1050) Same	0.037	0.042	0.048	0.051	0.056	0.063	0.072
ANOVA Bl. 4 (1051-2050) Same	0.031	0.037	0.046	0.050	0.055	0.065	0.076
ANOVA (FDR-BH) Bl. 1 (1-4) Δ_1	0.004	0.004	0.005	0.006	0.008	0.010	0.010
ANOVA (FDR-BH) Bl. 1 (5-350) Same	0.002	0.002	0.004	0.005	0.006	0.008	0.009
ANOVA (FDR-BH) Bl. 2 (351-354) Δ_2	0.140	0.140	0.144	0.148	0.151	0.155	0.155
ANOVA (FDR-BH) Bl. 2 (355-700) Same	0.004	0.005	0.007	0.008	0.009	0.010	0.012
ANOVA (FDR-BH) Bl. 3 (701-704) Δ_3	0.580	0.581	0.588	0.593	0.597	0.602	0.603
ANOVA (FDR-BH) Bl. 3 (705-1050) Same	0.002	0.003	0.005	0.006	0.006	0.008	0.009
ANOVA (FDR-BH) Bl. 4 (1051-2050) Same	0.000	0.000	0.000	0.001	0.002	0.003	0.006

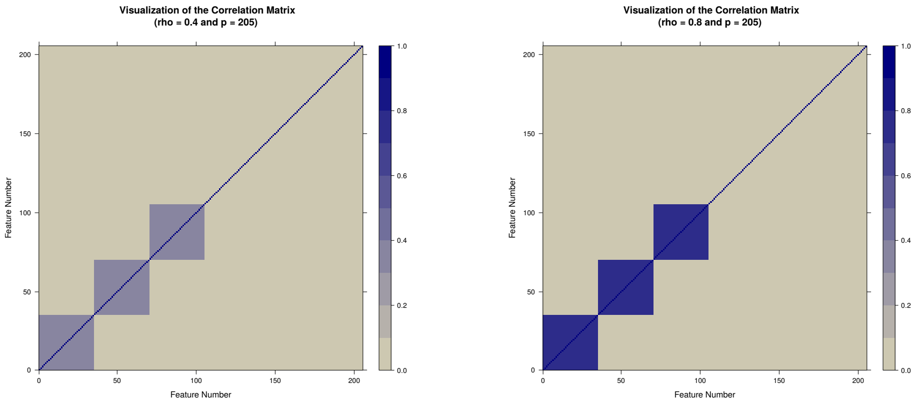

Figure A. Visualization of the dependency structure for the simulated data. Features are simulated in three blocks, where the correlation within each block is either $\rho = 0.4$ (left panel) and $\rho = 0.8$ (right panel). No dependency is simulated between blocks and as an independent set of features representing random noise is also simulated.

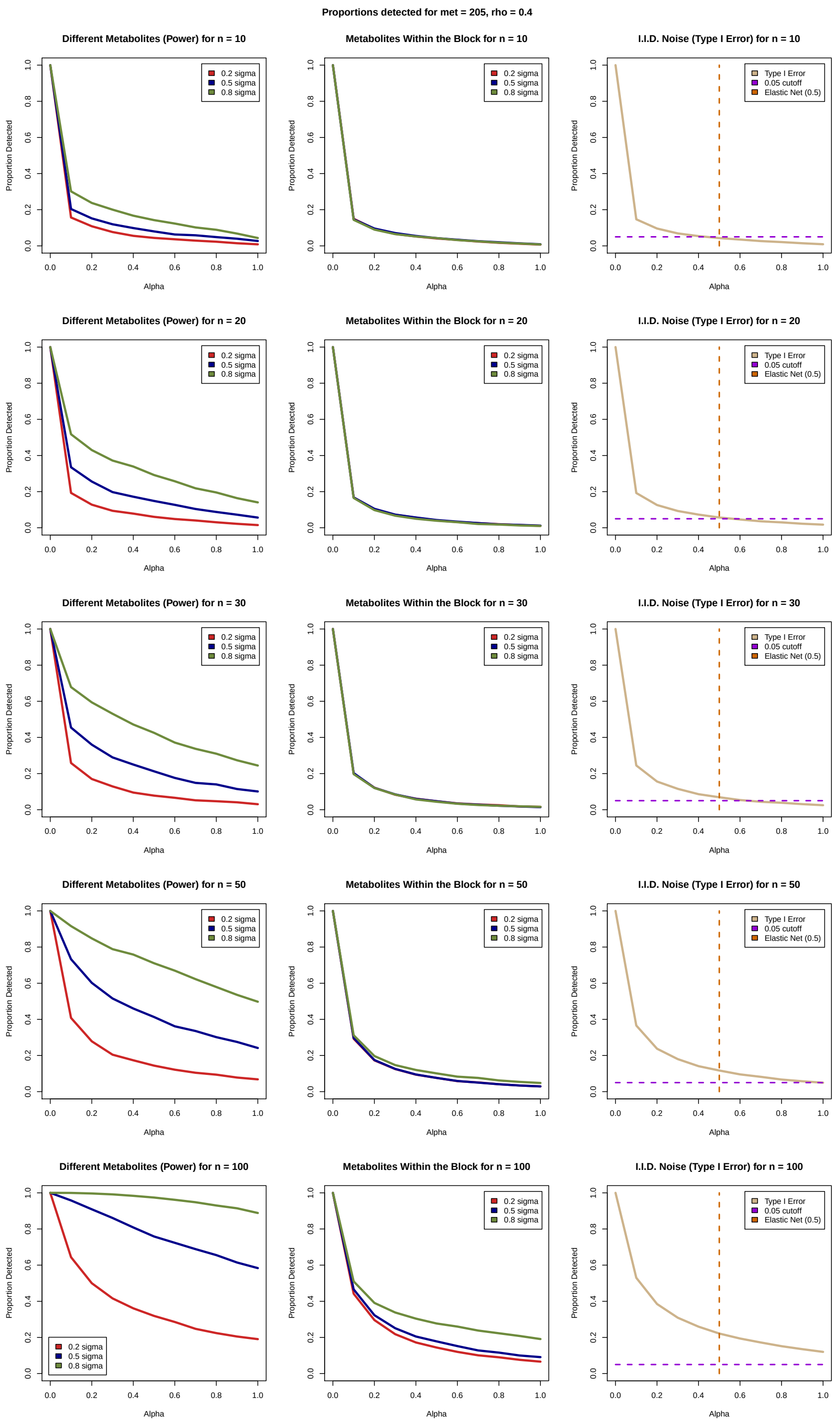
Proportions detected for met = 205, rho = 0.4
Different Metabolites (Power) for n = 10
Metabolites Within the Block for n = 10
I.I.D. Noise (Type I Error) for n = 10
Different Metabolites (Power) for n = 20
Metabolites Within the Block for n = 20
I.I.D. Noise (Type I Error) for n = 20
Different Metabolites (Power) for n = 30
Metabolites Within the Block for n = 30
I.I.D. Noise (Type I Error) for n = 30
Different Metabolites (Power) for n = 50
Metabolites Within the Block for n = 50
I.I.D. Noise (Type I Error) for n = 50
Different Metabolites (Power) for n = 100
Metabolites Within the Block for n = 100
I.I.D. Noise (Type I Error) for n = 100
Proportion Detected
Alpha
0.2 sigma
0.5 sigma
0.8 sigma
Type I Error
0.05 cutoff
Elastic Net (0.5)

Figure B. Visualization of power (left column) and Type I error (right column) estimates for $p = 205$ features, $\rho = 0.4$, and multiple sample sizes. Each row of plots corresponds to different sample size. The value of the penalty split parameter α is plotted on the x-axis. Type I error and power estimates are plotted on y-axis for the values of α in the range of $[0; 1]$ with 0.1 increments. In the left column power estimates are provided based on the four different features for each of the effect sizes ($\Delta_1 = 0.2$ is the red line, $\Delta_2 = 0.5$ is the blue line, and $\Delta_3 = 0.8$ is the green line). In the right column Type I error estimates are provided (beige lines) based on the random noise features together with a 0.05 threshold plotted as a purple dashed line. The vertical dashed line in the right column plots corresponds to penalty split value $\alpha = 0.5$. In the middle column of the plots the proportions of detected non-different features within each block correlated to different ones for each of the blocks and corresponding effect sizes ($\Delta_1 = 0.2$ is the red line, $\Delta_2 = 0.5$ is the blue line, and $\Delta_3 = 0.8$ is the green line) are displayed.

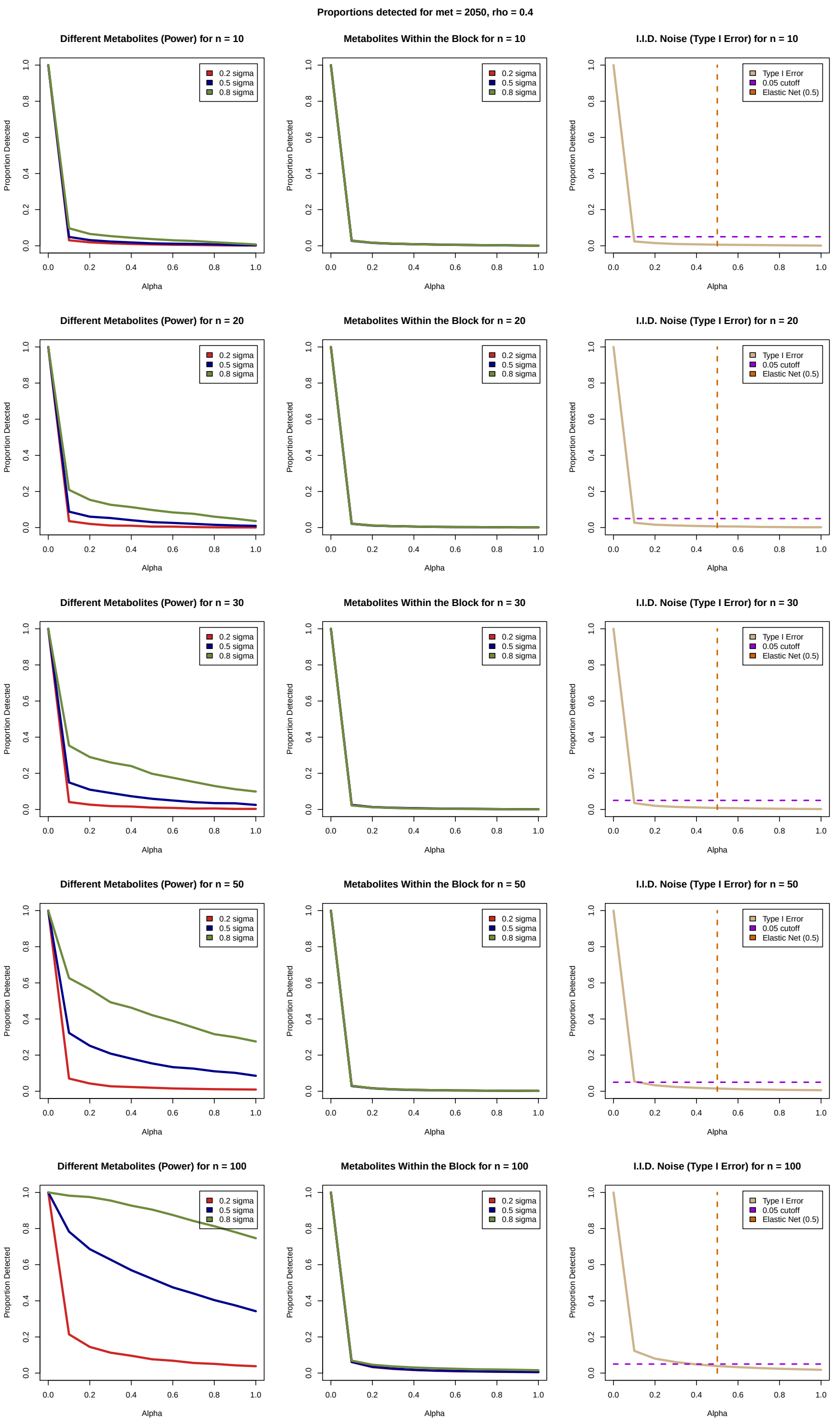
Proportions detected for met = 2050, rho = 0.4
Different Metabolites (Power) for n = 10
Metabolites Within the Block for n = 10
I.I.D. Noise (Type I Error) for n = 10
Different Metabolites (Power) for n = 20
Metabolites Within the Block for n = 20
I.I.D. Noise (Type I Error) for n = 20
Different Metabolites (Power) for n = 30
Metabolites Within the Block for n = 30
I.I.D. Noise (Type I Error) for n = 30
Different Metabolites (Power) for n = 50
Metabolites Within the Block for n = 50
I.I.D. Noise (Type I Error) for n = 50
Different Metabolites (Power) for n = 100
Metabolites Within the Block for n = 100
I.I.D. Noise (Type I Error) for n = 100
0.2 sigma
0.5 sigma
0.8 sigma
Type I Error
0.05 cutoff
Elastic Net (0.5)
Proportion Detected
Alpha

Figure C. Visualization of power (left column) and Type I error (right column) estimates for $p = 2050$ features, $\rho = 0.4$, and multiple sample sizes. Each row of plots corresponds to different sample size. The value of the penalty split parameter α is plotted on the x-axis. Type I error and power estimates are plotted on y-axis for the values of α in the range of $[0; 1]$ with 0.1 increments. In the left column power estimates are provided based on the four different features for each of the effect sizes ($\Delta_1 = 0.2$ is the red line, $\Delta_2 = 0.5$ is the blue line, and $\Delta_3 = 0.8$ is the green line). In the right column Type I error estimates are provided (beige lines) based on the random noise features together with a 0.05 threshold plotted as a purple dashed line. The vertical dashed line in the right column plots corresponds to penalty split value $\alpha = 0.5$. In the middle column of the plots the proportions of detected non-different features within each block correlated to different ones for each of the blocks and corresponding effect sizes ($\Delta_1 = 0.2$ is the red line, $\Delta_2 = 0.5$ is the blue line, and $\Delta_3 = 0.8$ is the green line) are displayed.

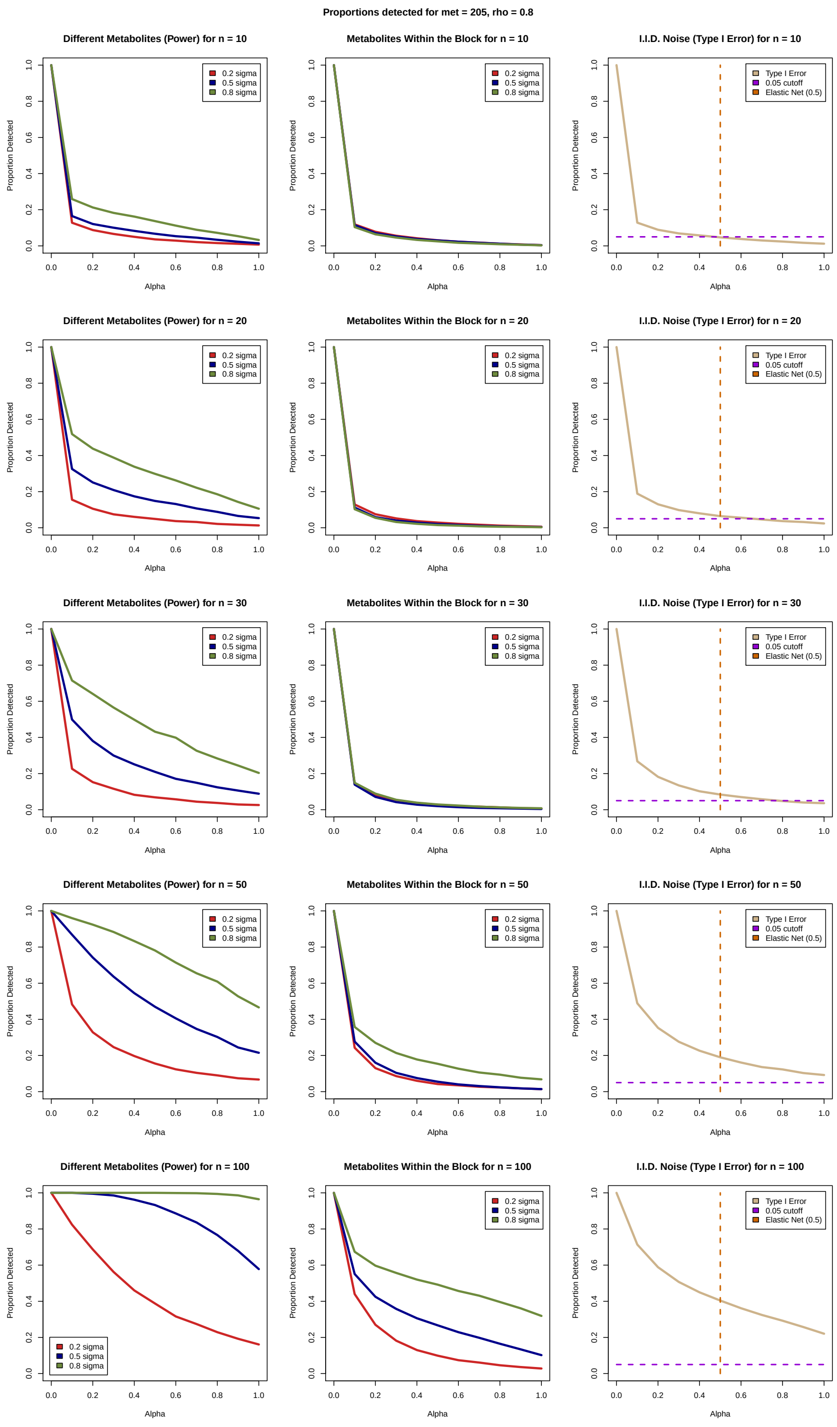
Proportions detected for met = 205, rho = 0.8
Different Metabolites (Power) for n = 10
Metabolites Within the Block for n = 10
I.I.D. Noise (Type I Error) for n = 10
Different Metabolites (Power) for n = 20
Metabolites Within the Block for n = 20
I.I.D. Noise (Type I Error) for n = 20
Different Metabolites (Power) for n = 30
Metabolites Within the Block for n = 30
I.I.D. Noise (Type I Error) for n = 30
Different Metabolites (Power) for n = 50
Metabolites Within the Block for n = 50
I.I.D. Noise (Type I Error) for n = 50
Different Metabolites (Power) for n = 100
Metabolites Within the Block for n = 100
I.I.D. Noise (Type I Error) for n = 100
Proportion Detected
Alpha
0.2 sigma
0.5 sigma
0.8 sigma
Type I Error
0.05 cutoff
Elastic Net (0.5)

Figure D. Visualization of power (left column) and Type I error (right column) estimates for $p = 205$ features, $\rho = 0.8$, and multiple sample sizes. Each row of plots corresponds to different sample size. The value of the penalty split parameter α is plotted on the x-axis. Type I error and power estimates are plotted on y-axis for the values of α in the range of $[0; 1]$ with 0.1 increments. In the left column power estimates are provided based on the four different features for each of the effect sizes ($\Delta_1 = 0.2$ is the red line, $\Delta_2 = 0.5$ is the blue line, and $\Delta_3 = 0.8$ is the green line). In the right column Type I error estimates are provided (beige lines) based on the random noise features together with a 0.05 threshold plotted as a purple dashed line. The vertical dashed line in the right column plots corresponds to penalty split value $\alpha = 0.5$. In the middle column of the plots the proportions of detected non-different features within each block correlated to different ones for each of the blocks and corresponding effect sizes ($\Delta_1 = 0.2$ is the red line, $\Delta_2 = 0.5$ is the blue line, and $\Delta_3 = 0.8$ is the green line) are displayed.

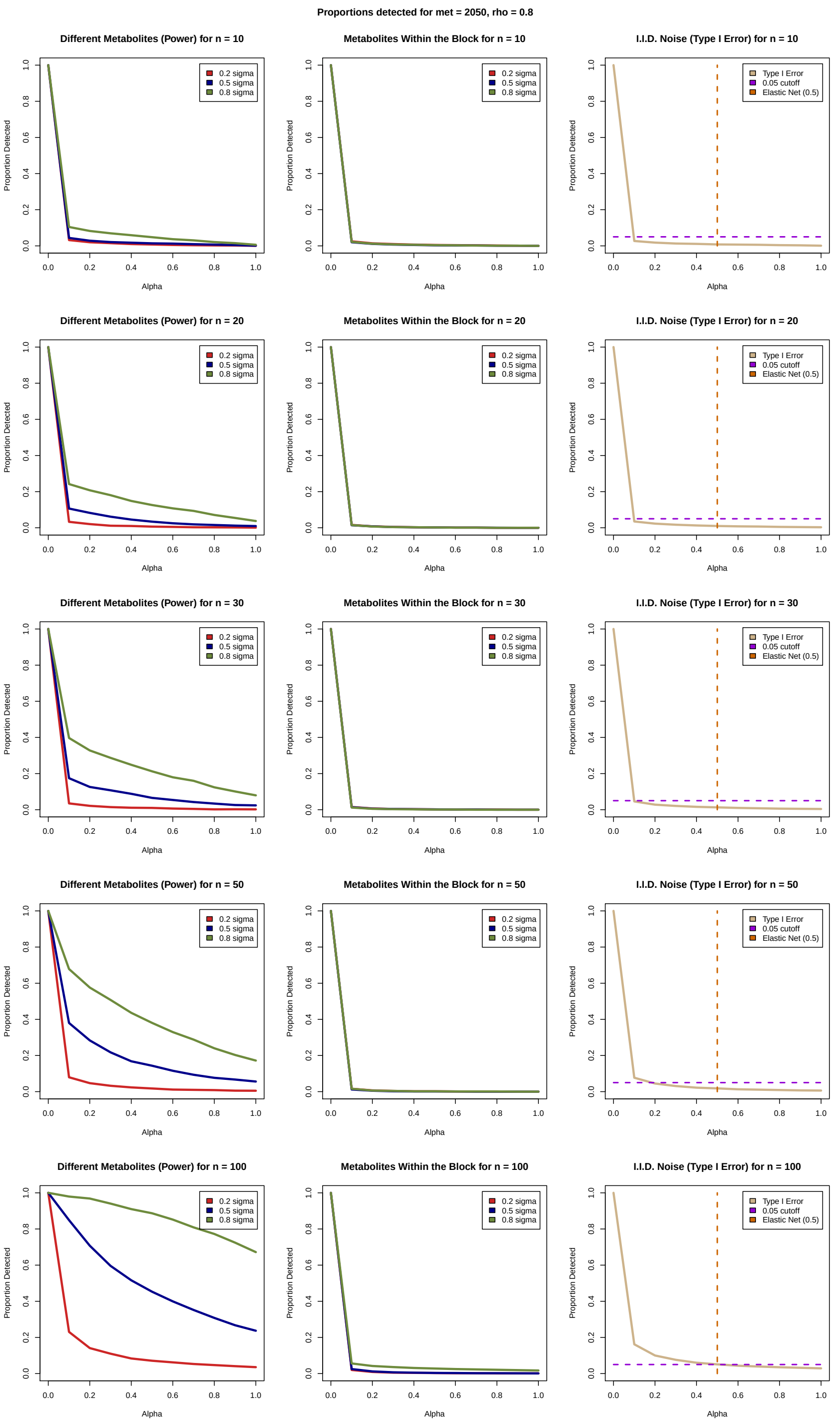
Proportions detected for met = 2050, rho = 0.8
Different Metabolites (Power) for n = 10
Metabolites Within the Block for n = 10
I.I.D. Noise (Type I Error) for n = 10
Different Metabolites (Power) for n = 20
Metabolites Within the Block for n = 20
I.I.D. Noise (Type I Error) for n = 20
Different Metabolites (Power) for n = 30
Metabolites Within the Block for n = 30
I.I.D. Noise (Type I Error) for n = 30
Different Metabolites (Power) for n = 50
Metabolites Within the Block for n = 50
I.I.D. Noise (Type I Error) for n = 50
Different Metabolites (Power) for n = 100
Metabolites Within the Block for n = 100
I.I.D. Noise (Type I Error) for n = 100
0.2 sigma
0.5 sigma
0.8 sigma
Type I Error
0.05 cutoff
Elastic Net (0.5)
Proportion Detected
Alpha
0.0
0.2
0.4
0.6
0.8
1.0

Figure E. Visualization of power (left column) and Type I error (right column) estimates for $p = 2050$ features, $\rho = 0.8$, and multiple sample sizes. Each row of plots corresponds to different sample size. The value of the penalty split parameter α is plotted on the x-axis. Type I error and power estimates are plotted on y-axis for the values of α in the range of $[0; 1]$ with 0.1 increments. In the left column power estimates are provided based on the four different features for each of the effect sizes ($\Delta_1 = 0.2$ is the red line, $\Delta_2 = 0.5$ is the blue line, and $\Delta_3 = 0.8$ is the green line). In the right column Type I error estimates are provided (beige lines) based on the random noise features together with a 0.05 threshold plotted as a purple dashed line. The vertical dashed line in the right column plots corresponds to penalty split value $\alpha = 0.5$. In the middle column of the plots the proportions of detected non-different features within each block correlated to different ones for each of the blocks and corresponding effect sizes ($\Delta_1 = 0.2$ is the red line, $\Delta_2 = 0.5$ is the blue line, and $\Delta_3 = 0.8$ is the green line) are displayed.

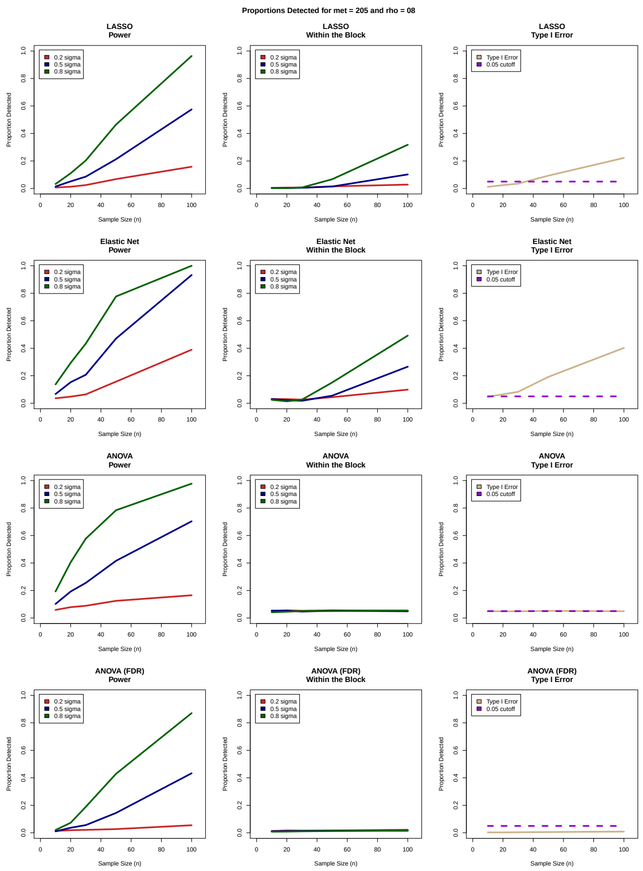

Figure F. Visualization of power and Type I error estimates comparison for $p = 205$ features, correlation $\rho = 0.8$, and all sample sizes. Each row of the plots corresponds to a feature selection method. ANOVA FDR adjustment cutoff is 0.2. The value of the sample size (n) is displayed on the x-axis in all plots. The estimates of power and Type I error are provided on the y-axis. In the left column power estimates are provided based on the four different features for each of the effect sizes ($\Delta_1 = 0.2$ is the red line, $\Delta_2 = 0.5$ is the blue line, and $\Delta_3 = 0.8$ is the green line). In the right column Type I error estimates are provided (beige lines) based on the random noise features together with a 0.05 threshold plotted as a purple dashed line. In the middle column the proportions of non-different detected features within each block correlated to different ones for each of the blocks and corresponding effect sizes ($\Delta_1 = 0.2$ is the red line, $\Delta_2 = 0.5$ is the blue line, and $\Delta_3 = 0.8$ is the green line) are displayed.

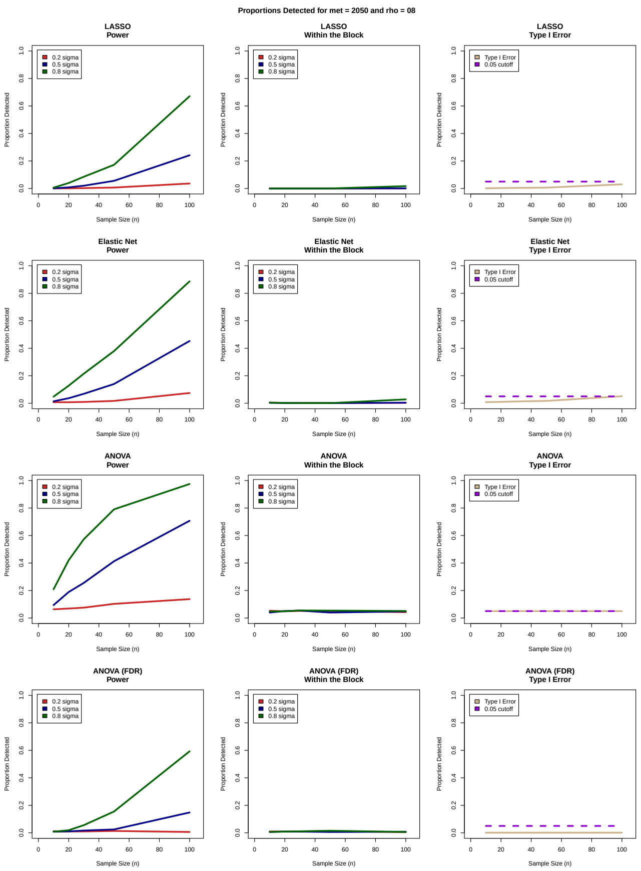

Figure G. Visualization of power and Type I error estimates comparison for $p = 2050$ features, correlation $\rho = 0.8$, and all sample sizes. Each row of the plots corresponds to a feature selection method. ANOVA FDR adjustment cutoff is 0.2. The value of the sample size (n) is displayed on the x-axis in all plots. The estimates of power and Type I error are provided on the y-axis. In the left column power estimates are provided based on the four different features for each of the effect sizes ($\Delta_1 = 0.2$ is the red line, $\Delta_2 = 0.5$ is the blue line, and $\Delta_3 = 0.8$ is the green line). In the right column Type I error estimates are provided (beige lines) based on the random noise features together with a 0.05 threshold plotted as a purple dashed line. In the middle column the proportions of non-different detected features within each block correlated to different ones for each of the blocks and corresponding effect sizes ($\Delta_1 = 0.2$ is the red line, $\Delta_2 = 0.5$ is the blue line, and $\Delta_3 = 0.8$ is the green line) are displayed.

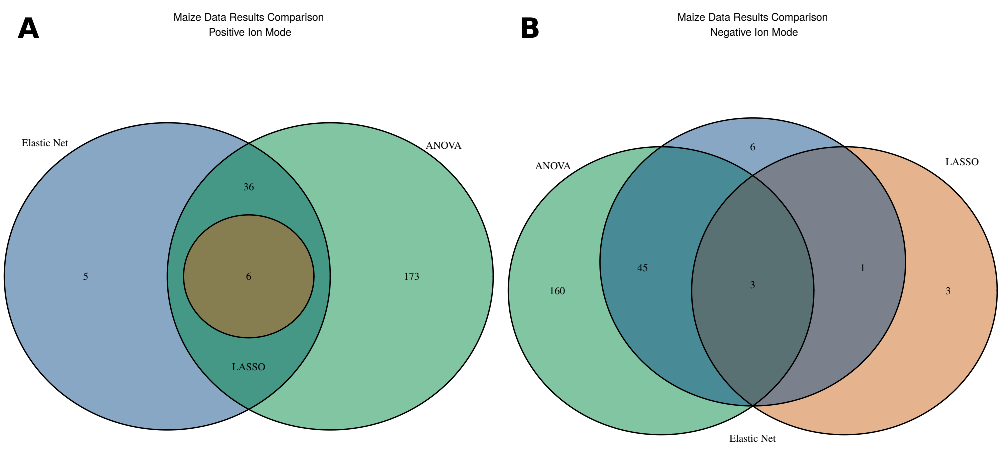

Figure H. Comparison of the variable selection methods for maize data using Venn diagrams. The results are provided for both positive and negative ion modes and for 0.05 cut off without adjustment for multiple testing. The results are provided in panels A) and B) for positive and negative modes respectively.

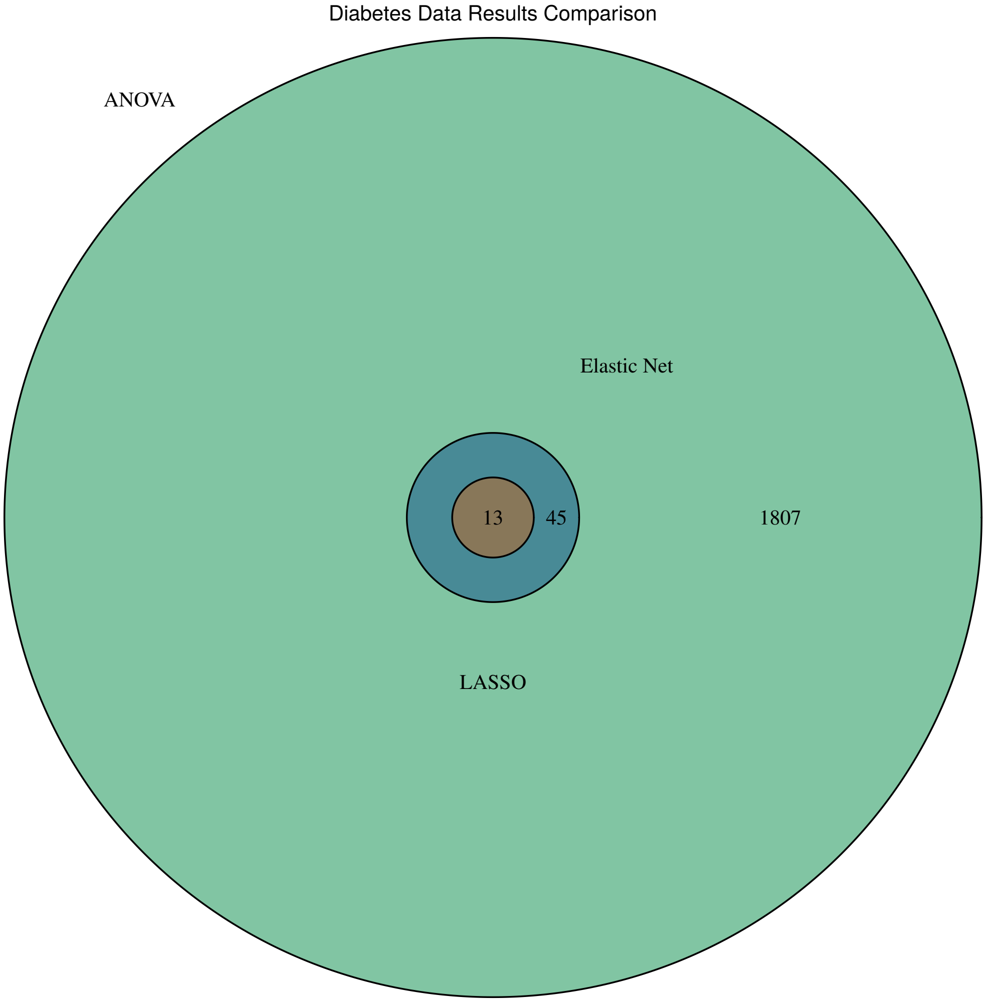

Figure I. Venn diagrams of the comparison of the variable selection methods for diabetes data. The results are presented for 0.05 cut off without FDR correction.

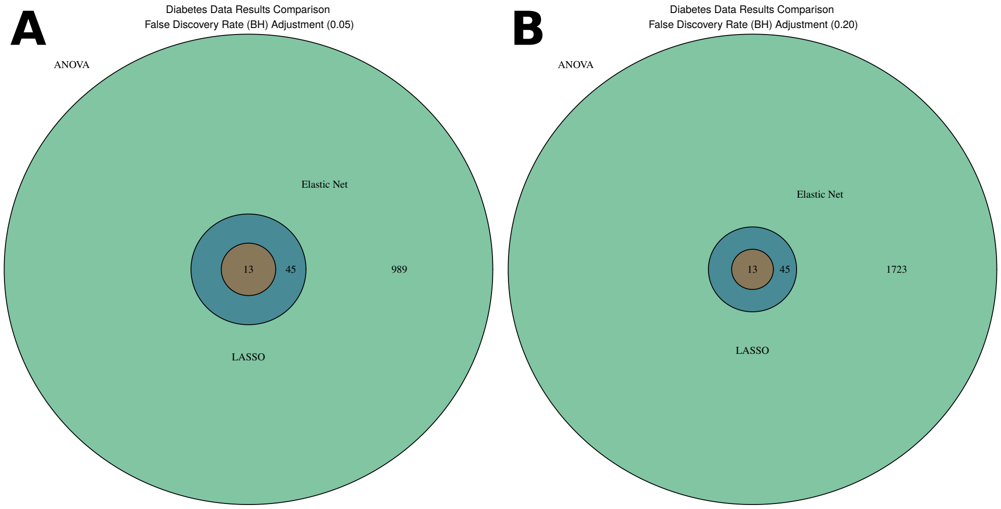

Figure J. Venn diagrams showing the results for the diabetes data. Panel A shows the overlap between ANOVA results with an FDR 0.05, LASSO and Elastic Net. Panel B shows the overlap between ANOVA results with an FDR 0.20, LASSO and Elastic Net. LASSO results are in brown and a subset of the Elastic Net results in Blue which are a subset of the ANOVA results shown in Green
